# Photomodulation of fluoride ion binding through anion-π interactions using a photoswitchable azobenzene system

**DOI:** 10.1038/srep22928

**Published:** 2016-03-08

**Authors:** Anushri Rananaware, Mousumi Samanta, Rajesh S. Bhosale, Mohammad Al Kobaisi, Biswajit Roy, Varun Bheemireddy, Sidhanath V. Bhosale, Subhajit Bandyopadhyay, Sheshanath V. Bhosale

**Affiliations:** 1 School of Applied Sciences, RMIT University, GPO Box 2476, Melbourne, VIC-3001, Australia; 2 Department of Chemical Sciences, Indian Institute of Science Education and Research Kolkata, Mohanpur, Nadia WB 741246, India; 3 Polymers and Functional Materials Division, CSIR-Indian Institute of Chemical Technology, Hyderabad, Telangana, 500007, India

## Abstract

The discovery of photoswitchable azobenzene-systems that undergo *trans-*to-*cis* photoisomerisation was a milestone in supramolecular chemistry. Such photoswitches have possible applications in data storage, stimuli responsive delivery systems, and molecular machines due to fast and selective switching. However, the light induced *cis* isomer of azobenzene is rather unstable and reverts thermally and photochemically to the thermodynamically stable *trans* configuration. We report, for the first time, controlled photoswitching of an azo-naphthalenediimide (azo-NDI) which can be achieved upon binding of fluoride ions through anion-π interaction. This NDI-F–NDI “sandwich” stabilises the *cis* configuration through the generation of an NDI^•−^ radical anion, and a dianionic, NDI^2−^ species that becomes unusually stable in the *cis* form. The sandwiched *cis* form reverts to the *trans* form only upon decomplexation of F^−^. A model pollutant was successfully degraded using the photogenerated NDI-F–NDI sandwich. This opens a wide range of applications in molecular and supramolecular nanotechnology.

Non-covalent interactions give rise to supramolecular assemblies with properties that can be applied in various fields of both science and engineering^1^. One of the challenges in designing supramolecular assembly is to produce systems that incorporate information and functions to drive the assembly towards practical devices at the molecular level that can be used, for example, in data storage devices^2^, sensors^3^, molecular machines^4^, and in life science applications^5^,^6^,^7^,^8^. During the last decade light induced molecular switches have gained enormous attention due to their achievement of fast and selective switching that can be exploited to elicit novel high gated function by these molecular switches^9^,^10^. Among the molecules used for switching, diarylethenes^11^, spiropyrans^12^,^13^ and azobenzenes^14^,^15^,^16^,^17^,^18^ are well-known. In most of these reported cases the *cis*-to-*trans* switching of azobenzene photoisomers in both directions occur reversible in multiple cycles with no chemical degradation, making the azobenzene based scaffold a highly potential candidate for an effective molecular switch with convertible isomers^19^,^20^. This conversion can proceeded *via* either in-planar inversion or out of plane rotation around the N=N double bond^21^. However, the light induced *cis* isomer of azo-benzene can be rather unstable as it undergoes both thermal and light induced conversion to the more thermodynamically stable *trans* isomer. There have been effective approaches where the azobenzene is incorporated into macrocyclic scaffolds, which can have a dramatic influence on the switching mechanism due to the stabilisation of *cis* isomer caused by ring strain^22^. Such macrocyclic arrangements are of great interest to the field of molecular computing as they were found to self-assemble on surfaces^23^ or self-organise into supramolecular channels^24^. The challenge in oligo-azobenzene design is the number of azo bridges and structure of the moieties connected to the azo groups may result in several conformational and structural isomers, making the *cis*–*trans* switching more complicated.

Recently discovered anion-π interaction^25^, a non-covalent interaction between an electron rich anion and an electron deficient aromatic π-system with a strong positive quadrupole moments^26^, opened new avenues to sensing^27^ and molecular recognition^28^ of anions. Nevertheless, the anion-π interactions are prominent players in several chemical and biological processes which can also be used in designing highly selective ion receptors and channels^29^,^30^. Among several anions, the interactions of fluoride anion (F^−^) with π-systems were extensively studied, often using the naphthalenediimide (NDI) fluorophore. NDI is frequently chosen as the π-system since it can be easily functionalised, dissolved in the range of solvents and has tuneable fluorescence properties^31^,^32^,^33^,^34^. Typically, the recognition of fluoride ion takes place through an anion-π interaction involving step wise electron transfers from F^−^ to the electron deficient NDI units that is reflected in a distinctive colour changes^35^,^36^,^37^.

Inspired by the reports of anion-π interactions of the NDI units^38^, herein, we report an unprecedented example of an NDI based receptor appended to an azobenzene photoswitch that undergoes *trans* to *cis* photoisomerization, where the thermodynamically unstable *cis* isomer has been conferred high stability in the presence of F^−^ through the formation of stable anionic species of NDIs i.e. NDI^•−^ and NDI^2−^ that are stabilized by anion-π interactions.

## Results

**Figure 1:**
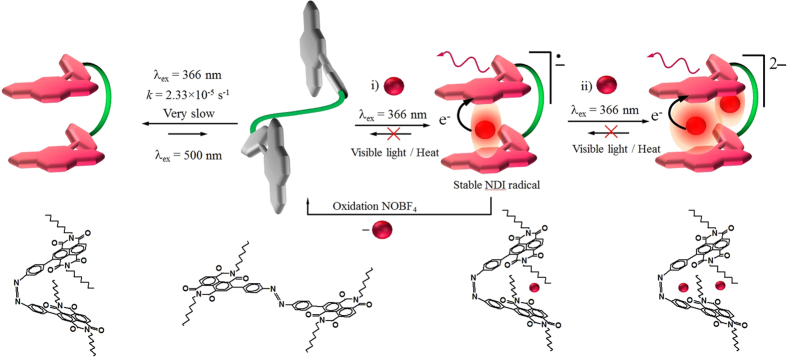
Graphical representation: of the F^−^ binding to the **azo-NDI** receptor in presence of UV light (366 nm) forming the *cis* radical anion in 1:1 fluoride to **azo-NDI** ratio in (**i**), and doubly charged complex with a 2:1 ratio in (**ii**). The electron transfer from F^−^ to **azo-NDI** resulted in fluorochromogenic effect and also an enhancement of the emission output. UV irradiated *trans*-**azo-NDI** in the presence of fluoride ions converts it to the more stable *cis* isomer through NDI/F^−^/NDI sandwich complex. In the absence of fluoride under UV irradiation (366 nm) *trans*-**azo-NDI** converted to the *cis* conformation, however this exchange is very slow in the order of k = 2.33 × 10^−5 ^S^−1^. Nevertheless, as expected *cis*-**azo-NDI** revert back to trans-form by irradiation with visible light (500 nm).

The azobenzene-NDI (**1**)–F^−^ complex can manipulated using photo control, as the *cis* form strongly interacts with F^−^ ions through a chromogenic anion-π interaction, which displays a distinct colour change upon binding (Fig. 1)^36^,^38^. The spectroscopic evidence including absorption, emission, ^1^H and ^19^F NMR, electrochemistry and EPR, suggest a two-step process in the formation of *cis*-**azo**-**NDI 1**–fluoride anion complex in the presence of UV light. The initial binding of F^−^ to **azo**-**NDI** generates an azo-NDI^•−^ radical anion that was detected using EPR spectroscopy. In the presence of excess F^−^ it forms doubly charged azo-NDI^2−^ anion which is EPR silent owing to its closed shell electronic structure.

In this work we aim to investigate the important aspects of the fluoride–**azo-NDI** binding including: 1) *trans*-to-*cis* isomerisation and the stability of the fluoride–**azo**-**NDI** species in the presence of fluoride ions, 2) the control and stability of the anion-π interaction in the *cis* isomer, 3) control of the switching behaviour *via* the stabilisation of the radical anion, 4) the role of the isomerisation played in the formation of supramolecular assemblies and 5) finally use of radical anions for degradation of pollutant (Rhodamine 123: Rh 123).

Detailed description of the synthesis of **azo**-**NDI** (**1**) and its intermediates are described in Methods and Supplementary Information.

### Photoisomerization

**Figure 2:**
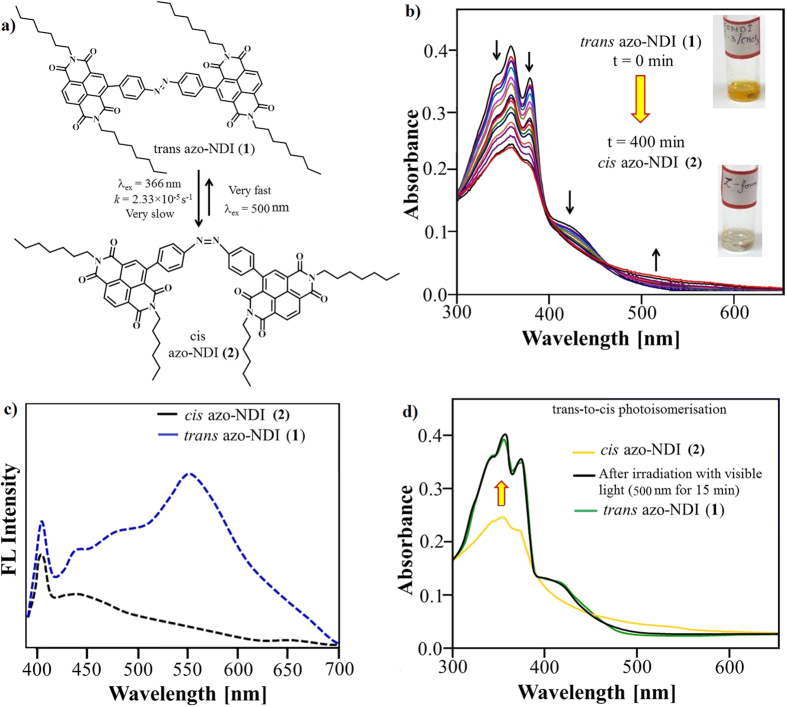
The photoisomerization of azo-NDI *trans* to *cis* and back. (**a**) The scheme of photoisomerization of **azo-NDI** *trans* to *cis* and back using 366 nm and 500 nm respectively. (**b**) The kinetic evolution of *cis* isomer from *trans* during UV irradiation at 366 nm and accompanied color change in the solution. (**c**) The fluorescence spectra of *cis* and *trans* **azo-NDI**. (**d**) The absorption of evolution of *cis- to trans-***azo-NDI** induce by irradiation at 500 nm visible light monitored using UV-vis spectroscopy.

The absorption spectra of **azo**-**NDI** (**1**) derivatives in DMF solution are depicted in Fig. 2, which exhibits two absorption bands in the UV region at 358 and 373 nm attributed to the π−π* (S_0_→S_2_) this transition showing two NDI chromophores are in different environment i.e. confirms trans configuration and a shoulder at 440 nm attributed to n-π* (S_0_→S_1_) electronic transitions. The **azo**-**NDI** (**1**) solution was irradiated with UV (366 nm) or visible light (500 nm) to probe the occurrence of light-induced *trans*–*cis* photoisomerization in DMF as illustrated in Fig. 2a. This process was monitored using UV-vis spectra (Fig. 2b). Under UV irradiation at 366 nm, a decrease in the π−π* absorption bands at 358 nm and 373 nm with an increase at broad band between 500–530 nm (of the n–π* band) along with isosbestic point at 489 nm was observed. The *trans* to *cis* photoisomerization reached a completion in 400 minutes with a rate constant of *k *= 2.33 × 10^−5 ^s^−1^. Upon photoisomerization from *trans* to *cis* gives significant sharp decrease in the strong π−π* bands with a molar extinction coefficient ε = ~ 5 × 10^4 ^M^−1^ cm^−1^ compared to the increase in the much weaker n→π* band with ε = ~ 300 M^−1^ cm^−1^. However, the π−π* bands in the *cis*-**azo-NDI** has a molar extinction coefficient of ε = ~ 9 × 10^3 ^M^−1^ cm^−1^, and n→π* band at 530 nm is allowed in the *cis* isomer an increase in molar extinction coefficient to ε = ~ 1500 M^−1 ^cm^−1^ with respect to the *trans* isomer resulting in the isomerization process being accompanied by a visual change to less intense colours.

The fluorescence (λ_ex_ = 360 nm) of trans **azo-NDI** (**1**) reveals rather broad peak at 550 nm with a quantum yield of Φ_F_ = 2.37% along with the sharp band at 401 nm, which is quenched in *cis* isomer of **azo-NDI** (**2**) where NDI moieties undergo π−π interaction within *cis*-**azo-NDI** (**2**) molecule as shown in Fig. 2c.

The UV-vis absorption spectroscopy illustrates close to zero thermal *cis*-to-*trans* isomerisation which may be due to structural restrictions in a rotating mechanism (ESI Fig. S1).

As expected, when a solution of *cis*-**azo-NDI** (**2**) was exposed to visible light, through a band pass filter 500 ± 17 nm, using handheld UV lamp (~1 mW cm^−2^), the reversal *cis*-to-*trans* isomerisation was observed *via* evolution of the π−π* absorption bands. The UV-vis absorption spectra clearly show *cis*-to-*trans* isomerization with matching absorption peaks to the original **azo-NDI** (**1**) within only 15 minutes (Fig. 2d), with a mechanism similar to that which observed for isomerization of stilbenes^39^.

The available data suggests that the isomerization of **azo-NDI** (**1**) proceeds through two different mechanisms; (i) the isomerisation from *trans*-to-*cis* passes through a rotating mechanism, and (ii) the thermal *cis*-to-*trans* isomerisation follows an inversion mechanism^40^, as the non-bonding electron pair of each nitrogen atom may lead to one n→π* electronic transition (S_0_→S_1_) with inversion at the nitrogen atom.

### NDI/F^−^/NDI sandwich complex

**Figure 3:**
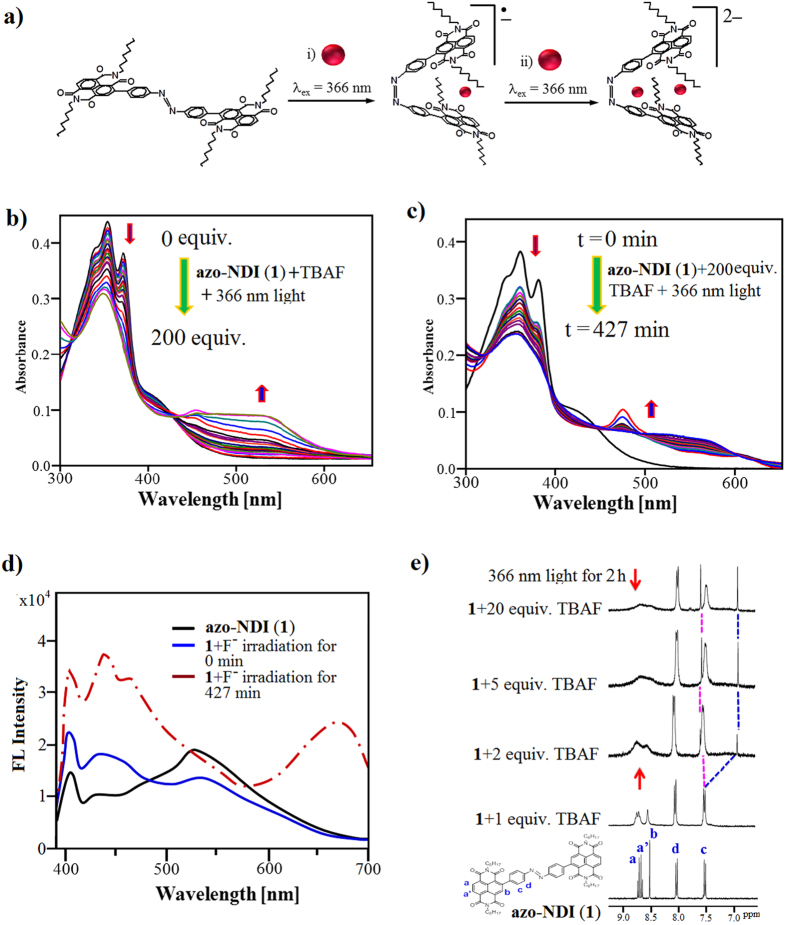
Anion-π interaction. (**a**) Schematic representation of the photoinduced complexation/isomerization of *trans*-**azo-NDI** (**1**) to form the F^−^ -*cis*-**azo-NDI** and 2F–*cis-***azo-NDI** complex under UV 366 nm laser irradiation. (**b**) The evolution of UV-vis spectra of **azo-NDI** at increasing fluoride ion ratios. (**c**) The evolution of UV-vis spectra of **azo-NDI** with time at 1:200 fluoride ion ratio during the complex formation under UV irradiation (366 nm). (**d**) The fluorescence spectra of *trans*-**azo-NDI** (**1**) and in the presence of fluoride ion with irradiation of 0 and 427 min, respectively. (**e**) ^1^H-NMR of **azo-NDI** at various ratios of fluoride ion.

**Figure 4:**
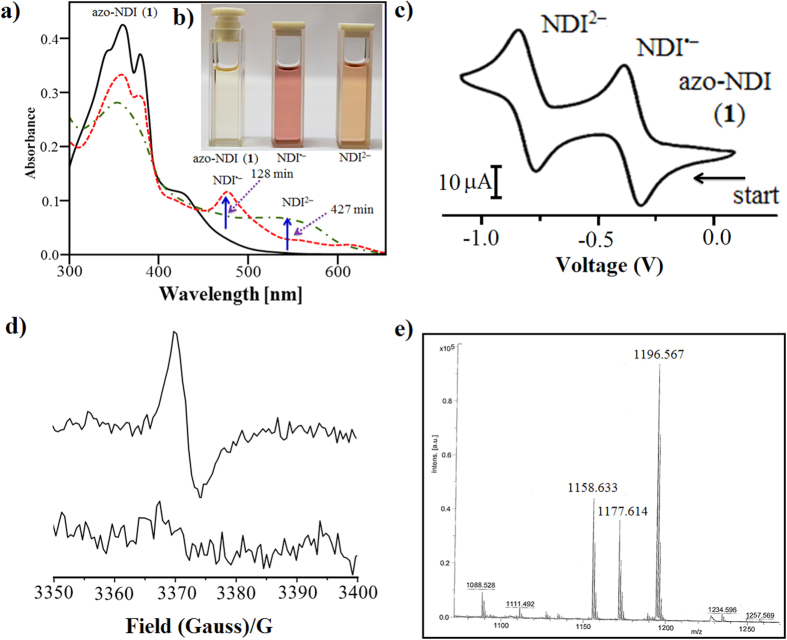
F^−^-azo-NDI complex. (**a**) The UV-vis spectra (shown in “**a**”) at detected oxidation states. (**b**) Inset in “**a**” is visual colour change of azo-NDI at the native, radical ion and dianion states. (**c**) Cyclic voltammetry (CV) of compound azo-NDI (**1**) in DMF (2 mM) with 0.1 M tetrabutyl ammonium hexafluorophosphate (TBAPF_6_) supporting electrolyte. (**d**) Electron paramagnetic resonance (EPR) of the radical ion and dianion species of **azo-NDI**. (**e**) ESI-MS of *cis*-**azo-NDI** at the native, *cis*-**azo-NDI** with 1xF^−^ and with 2 × F^−^, respectively.

It is well known that electron deficient NDIs are capable of complexation with fluoride through anion-π interactions and generate the NDI^•−^ and NDI^2−^ species^35^,^36^. Therefore, **azo-NDI** (**1**) was irradiated with UV (366 nm) in presence of F^−^ (TBA salt) with aim of probing the occurrence of light-induced *trans*-to-*cis* photoisomerization in DMF through F^−^–NDI CT complexation (Fig. 3a). UV-vis absorption spectra presented in Fig. 3b, the **azo-NDI** (**1**) (6 μM) absorption peaks gradually decreases at 358 and 373 nm and simultaneously produced new band at 476 nm upon titration of 0–4 equiv. of F^−^ (TBA salt). Further addition of F^−^ (5–200 equiv.), the absorption peak at 476 nm gradually vanishes with the appearance of a broad absorption band at 560 nm. Thus, *trans*-**azo-NDI** (**1**) is completely converted into *cis*-**azo-NDI** (**2**) through anion-π interaction forming NDI/F^−^/NDI sandwich complex as illustrated in Fig. 4a. Despite anions (F^−^, Cl^−^, Br^−^, I^−^, AcO^−^, and H_2_PO_4_^−^) being in excess (200 equiv.) only F^−^ formed NDI/F^−^/NDI sandwich complex (ESI Fig. S2).

Furthermore, *trans*-to-*cis* photoisomerisation of **1** was evaluated by changes in absorption in the presence of F^−^ over the time period (Fig. 3c). The **azo-NDI** (**1**) (6 μM) with 1.2 mM F^−^ (TBA salt) was mixed together in cuvette and time dependent absorption changes were monitored by irradiating sample with UV light. It showed that over the time π−π* **azo-NDI** (**1**) peaks at 358 and 373 nm decreased and simultaneously produced new band at 476 nm, with clear isosbestic point at 443 nm within 128 min and the solution turned violet in colour. Upon continuous irradiation with a UV lamp over time (>128 min), the absorption band at 476 nm gradually vanished with the appearance of a broad absorption band at 560 nm with a clear isosbestic point at 495 nm. Similarly, photoisomerisation of *trans*-to-*cis* also occurred in the presence of different fluoride source i.e. KF salt as shown in ESI Fig. S3. The overlay of the cis and the cis + F^−^ clearly differentiates between *cis*-**azo-NDI** and formation of NDI radical and dianions in the presence of F^−^ anions, respectively (ESI Fig. S4).

The fluorescence of receptor **1** displays a peak at 401 nm and a broad peak at 546 nm (Fig. 3d). Upon the addition of F^−^ and irradiation under UV, the sample’s fluorescence peak at 401 nm increased along with the appearance of new peaks at 440 and 546 nm at 0 min. Continuous irradiation of the sample for >400 min, a new florescence peaks appeared at 689 nm along with increased in intensity of the original peaks at 401 and 440 nm. Thus, the high F^−^ ion sensitivity of organized receptors azo-NDI sandwich suggests that it can be applied as F^−^ ion sensor. The fluorescence quantum yield also increased to *Ф*_*f*_ = 14.98% upon binding of fluoride – ***cis*****-azo-NDI** (**2**) complex formation, which can be attributed to aggregation induced emission (AIE) effect^41^. Higher *Φ*_*f*_ is expected from such complex, however, due to strong irradiation during the process of *trans-*to*-cis* conversion of **azo-NDI** (**1**) it is severely quenched the emission by annihilating the radical ions.

To gain further insight into NDI/F^−^ binding event, ^1^H-NMR titrations were carried out by addition of TBAF to **1** upon irradiation with UV light for 2 h (Fig. 3e). The ^1^H-NMR spectra of **azo-NDI** (**1**) displays a singlet at 8.57 ppm, and doublet of doublets (dd) at 8.76–8.93 ppm corresponding to H_b_ and H_a,a’_ of the NDI core proton, also two doublets at 7.50 and 8.05 ppm corresponding to H_c_ and H_d_ of the aryl proton. Upon the addition of 1 equiv. of TBAF all the NDI core protons at 8.57 and 8.76–8.93 ppm broaden which confirms anion-π interactions between NDI core and F^−^ anion. Further addition of TBAF (2 to 20 equiv.) results in a second fluoride intercalation between the aryl moieties connected to the azo-group, which was confirmed by the broadening of H_c_ proton followed by splitting of this peak into two peaks at 6.98 and 7.63 ppm along with further broadening of the peaks relating to the NDI core protons. These results also confirm that upon 1 equiv. addition of F^−^, NDI^•−^ is formed and further addition causes the formation of 2F^−^–*cis-***azo-NDI** complex. The NDI/F^−^/NDI sandwich complexation was further supported by the ^19^F NMR spectroscopy with and without UV irradiation (ESI Fig. S5a,b, respectively).

The absorption band (Fig. 4a) appeared at 476 nm over 128 min perfectly matches with that of an electrochemically generated NDI^•−^ radical anion (−450 mV vs Ag/AgCl in DMF) produced in the absence of F^−^ as illustrated in Fig. 4c. Furthermore, irradiation with UV laser (366 nm) for longer times i.e. 128–400 min, the absorption band at 476 nm of NDI^•−^ disappeared with appearance of a new broad band at 560 nm, which is similar to electrochemically generated NDI^2−^ species at −890 mV vs Ag/AgCl in DMF in the absence of F^−^36. Thus, the formed *cis***-azo-NDI** (**2**) sandwich complex was stabilised through charge transfer (CT) and the formation of NDI^•−^ radical anion and NDI^2−^ dianion.

The formation of NDI^•−^ and NDI^2−^ resulting from host–guest charge transfer complex (CT) of F^−^ ion and *cis*-**azo-NDI** (**2**) can be visually observed (inset Fig. 4b in 4a)^36^. Typically, **azo-NDI** (**1**) in DMF gives pale yellow colour upon addition of 4 equiv. of TBAF, during irradiation with UV light (366 nm) the pale yellow colour of the solution changes to violet in ~128 min, further irradiation or addition of >5 equiv. of F^−^ resulted violet to orange colour.

The electron paramagnetic resonance (EPR) spectrum of F^−^ induced violet solution of **1** upon irradiation at 366 nm further confirms the formation of a delocalized NDI^•−^ radical anion (*g* = 2.0038) as shown in Fig. 4d. These results attributed formation of anion-π interactions through F^−^→NDI electron transfer and the generation of NDI^•−^ *via* formation of NDI/F^−^/NDI sandwich complex. Upon irradiation of F^−^→NDI complex for more than 130 min the color of solution turned from violet to orange which is consistent with the formation of NDI^2−^, as EPR became silent. The F radical species sandwiched by the NDI radical anion is stabilized by strong electrostatic interaction. These are not photoinduced excited states, otherwise the species cannot be long lived.

The formation of NDI/F^−^/NDI sandwich dianion was also evident from the electrospray ionization mass spectrometry (ESI-MS) recorded in positive-ion mode (Fig. 4e and Fig. S6). The m/z peak at ~1158 corresponding to azo-NDI, peak at 1177 correspond to cis-NDI/F^−^/NDI sandwich complex and m/z peak at 1198 matches with NDI/2F/NDI^]2−^, respectively. ESI-MS in negative ion mode shows two typical peaks that correspond to the mass of the NDI^2−^ of *cis-***azo-NDI** (**2**) receptor associated with H^+^ and Na^+^ ions, respectively (Supplementary Information Fig. S7). Thus, ESI-MS spectroscopy results confirm formation of NDI/F^−^/NDI sandwich complex.

**Figure 5:**
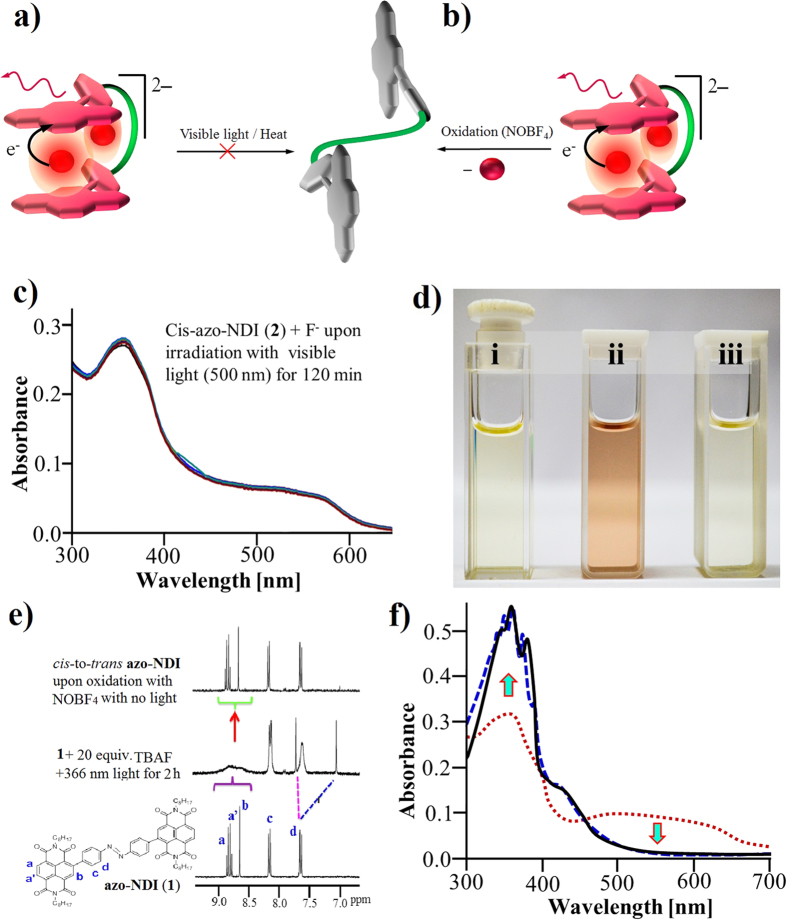
The reversibility of the NDI/F^−^ complexes. (**a**,**b**) Schematic representation of the complex oxidation process using NOBF_4_ to produce *trans*-**azo-NDI** while this process do not proceed *via* heat or irradiation using light above wavelengths of 500 nm. (**c**) The UV-vis spectra change after irradiating the complex solution using visible light with 500 nm cut off for 120 min. (**d**) Is visual colour change of *trans*-**azo-NDI** at the native (i) radical ions (ii) and decolourisation to the original *trans*-**azo-NDI** native color (iii), shows the reverse system *cis*-to-*trans* after oxidation using NOBF_4_. (**e**) The ^1^H-NMR spectra of the initial *trans*-azo-NDI and NDI/F^−^ complexes at 1:20 equiv. ratio of NDI:TBAF upon irradiation with UV light at 366 nm and the fully recovered *trans*-**azo-NDI** after oxidation using NOBF_4_. (**f**) The UV-vis spectra after oxidation using NOBF_4_ and the formation of *trans*-**azo-NDI**.

Interestingly, the NDI radicals and anions that formed were shown to be very stable (Fig. 5a,b), as observed changes are not fully reversed even upon irradiation with visible light (500 nm cut of light) for hours (Fig. 5c) and also thermally for longer time (ESI Fig. S8). Notably, upon oxidation of both NDI^•−^ and NDI^2−^ stable species using NOBF_4_, the *cis*-to-*trans* conversion can be monitored by decolourisation of the solutions from orange colour to original pale yellow colour as shown in Fig. 5d. The ^1^H-NMR spectrum also confirmed complete recovery of **azo-NDI** (**1**) after oxidation, as the spectrum shows the reappearance of all the peaks i.e. H_a_, H_a’_, H_b_ and H_c_ which were broaden and shifted as shown in Fig. 5e . UV-vis absorption spectroscopy further confirms regeneration of original spectra of *trans*-**azo-NDI** upon oxidation (Fig. 5f). The reversible nature of the NDI/F^−^ interactions is further confirmed that NDI-F^−^ are non-covalent anion-π interactions and there is no C–F bond formation. The fluorescence spectrum also confirms these interactions.

The interaction between F^−^ and the **azo-NDI** receptor **1** without UV irradiation (366 nm) is weaker and kinetically less favourable (ESI Fig. S9a–d) and does not show any interactions with anions other than F^−^ (ESI Fig. S10).

### Field Emission Scanning Electron Microscopy (FE-SEM)

**Figure 6:**
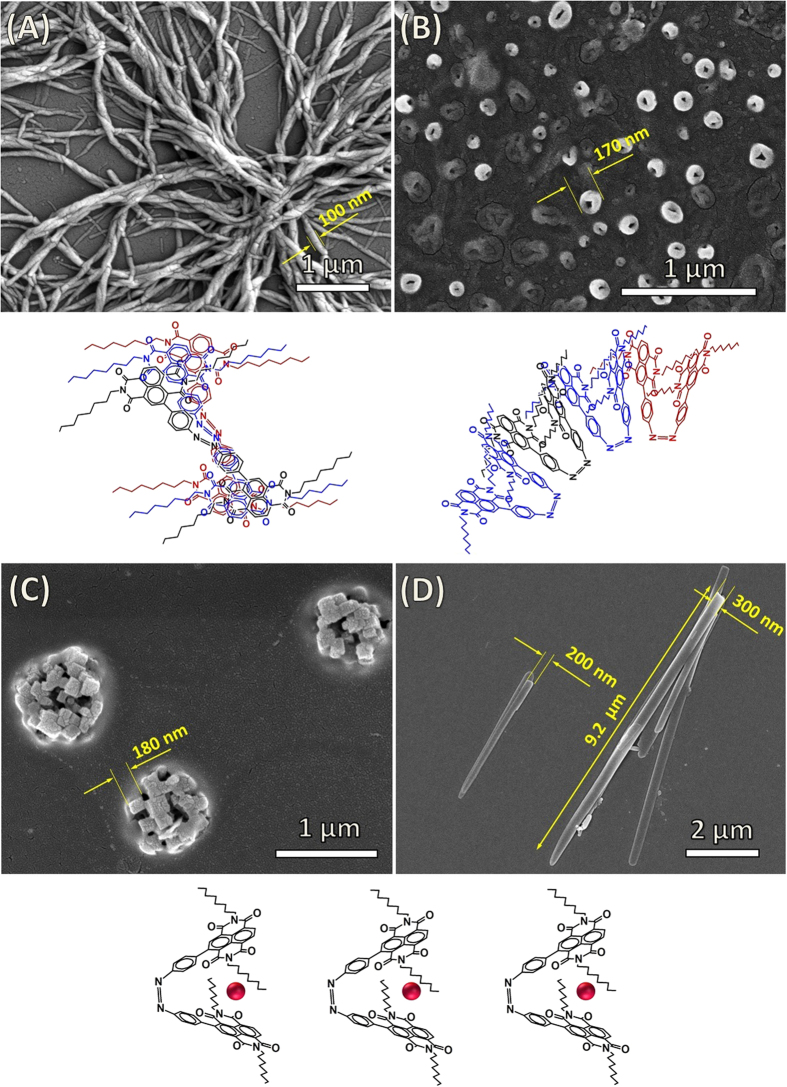
SEM micrographs of azo-NDI assemblies at various conditions. (**A**) Twisted and bundled nano-fibers from *trans* isomer before association with fluoride ion and irradiation. (**B**) Vesicles produced by *cis* isomer upon irradiation using 366 nm in the UV region. (**C**) Cubic crystalline assembly observed when **azo-NDI** is associated with fluoride ion without irradiation. (**D**) Micro-belt when associated with fluoride ion upon irradiated with UV light (366 nm).

The self-assembly of **azo-NDI** (**1**) under various conditions, which gave the molecule various geometries and electronic structures were studied using electron microscopy of the molecular aggregates that were deposited on a silicon wafer substrate (Fig. 6)^42^. The *trans*-**azo-NDI** isomer gave a twisted long fibril structures 10–150 nm in diameter, Fig. 6A, these structures mainly formed due to the solvophobic effects especially when the *trans*-**azo-NDI** molecule is solubilized in water, resulting in the prominence of hydrophobic interactions between the large hydrophobic alkyl groups and the π−π stacking of NDI and the other aromatic moieties as shown schematically in Fig. 6. The *cis* isomer forms vesicles, which is consistent with the asymmetric and the more polarized geometry of the isomer, as shown in Fig. 6B and the scheme below. Formation of the ionic species in the presence of fluoride ions brings on the stronger ionic interactions in to actions, results in more crystalline aggregates. SEM micrographs show cubic nano-crystalline formation about 180 nm in dimension, Fig. 5c and scheme below. The UV-irradiated (366 nm) (C) resulted in the directional growth of the crystalline aggregates into high aspect ratio belts 200–300 nm wide and many microns in length, Fig. 6D.

The formation of aggregates in solution was confirmed by dynamic light scattering (DLS) analysis of **1** and **2** (1 × 10^−4 ^M) as shown in ESI Fig. S13. The *cis* conformation result in smaller aggregate with a hydrodynamic diameter in the range of 20–100 nm, DLS also shows a shoulder extending to diameters bellow 700 nm produced by the coagulation of the primary small aggregates. This is in agreement with the SEM micrographs showing vesicle aggregates similar in the size distribution. The *R*H of the vesicles derived from the characteristic line width were obtained by the CONTIN analysis method^43^. The *cis* isomer in its salt form, produced by the addition of TBAF, shows a modulated size distribution in the DLS measurement, where the smallest aggregates are 50–200 nm in hydrodynamic diameter. These primary aggregates grow further to produce particles up to >2 μm hydrodynamic diameter. The SEM images confirm this observation showing needles like crystalline particles up to 300 nm in width which has grown further in length upon solvent evaporation.

The *trans* isomer of azo-NDI produced aggregates in solution with 100–700 nm in hydrodynamic diameter. The SEM images of these aggregates, Fig. 6(A), after solvent evaporation shows that these aggregates are fibre like and can grow larger length with increased concentration. The salt of the *trans* isomer of azo-NDI after the addition of TBAF give smaller primary aggregates in the range 20–100 nm, these aggregates further coagulated to produce larger formation up to 1.1 μm in diameter as can be seen in Fig. 6(C) SEM micrograph.

### Pollutant degradation

Rhodamine dyes are often used in industrial fields due to its high stability^44^. However, if ingested, it causes irritation to skin, eyes and respiratory tract^45^. Using our photogenerated *cis* NDI^•−^ stable radical anion, we have studied the degradation of a model pollutant dye Rh-123. The degradation of Rh-123 dye was successfully demonstrated by the loss of the characteristic absorption peak at 517 nm of the Rh-123 solution in the presence of NDI^•−^ anions in <5 min (Figs S11 and S12).

### Discussion

We have shown that a dynamic system based on simple photoisomerisation in the presence of an anion guest, causes; 1) the stabilisation of *cis* configuration, 2) stable NDI radicals, and 3) system can be reversible in the presence of oxidising agent. The experimental support illustrates that NDI/F^−^ interactions facilitate an unprecedented F^−^→NDI electron transfer (ET), supported by generation of the stable NDI^•−^ radical species and further to a NDI^2−^ dianion. Along with absorption, electrochemistry and EPR spectroscopy confirms the formation of radical species through CT complex of NDI/F^−^/NDI anion–π interaction. Furthermore, the molecular modelling of rigid model of one HF molecule and an NDI core 2 Å apart using Hartree-Fock and 3–21 g level of theory shows that there is high electron density between the HF and the NDI core in the molecular orbitals between 88 and 99 states indicative of possible binding interaction between the two moieties as shows in Supplementary Information Figs S14 and S15. In the absence of an oxidising agent, the *cis* configuration of **azo-NDI** system is very stable even in presence of visible light/heat due to generation of stable NDI radicals. The cis form of the azo-system described here is so robust in the presence of the anion that it shuts off the photoisomerization to the *trans* form. The transformation to the trans form can only be achieved after the release of the fluoride guest *via* deconstruction of the *cis* form of **NDI/F**^**−**^**/NDI** sandwich system through an oxidation of the NDI radical species. Furthermore, supramolecular self-assembly provided very interesting results such as twisted and bundled nano-fibers from *trans* isomer before association with fluoride ion, however, vesicular aggregated produced by *cis* isomer upon irradiation (366 nm). Furthermore, cubic crystalline assembly formed when **azo-NDI** is associated with fluoride, and micro-belt when **azo-NDI** associated with fluoride ion and then irradiated using 366 nm. Finally, we explore use of NDI^•−^ anions for degradation of environmental pollutant (Rhodamine 123: Rh 123).

## Methods

### Materials

All the chemicals and reagents were purchased from Sigma Aldrich and used as received without further purification. Reactions were monitored by thin layer chromatography using Merck plates (TLC Silica Gel 60 F254). For the separation of compounds in column chromatography 100–200 mesh silica gel were used. The structures of the compound were confirmed by ^1^H-NMR and ^13^C-NMR and other spectroscopic techniques. NMR spectra were recorded in 400 MHz Jeol and 500 MHz Bruker spectrometers in appropriate deuterated solvents. Chemical shifts were recorded in δ value using either residual solvent signals or tetramethylsilane as internal standards. Mass data were obtained from an Acquity^TM^ ultra performance LC in ESI (−) ve and (+) ve modes. The solvents used in spectroscopic techniques were of spectroscopic grades and were free from any fluorescent impurities. The solutions of the anions were prepared from TBAF, TBACl, TBABr, TBAI, TBAOAc, TBAH_2_PO_4_ in DMF.

### UV-vis spectroscopy

UV-vis spectra were recorded with a Cary 60 UV-vis spectrophotometer. The receptor **1** undergoes *cis*–*trans* isomerisation in presence of 366 nm light. The peak at 358 gradually decreases and a new peak at 560 nm is being formed. The molecule in the *cis* form detects the fluoride ion in a twostep process. In the first step it forms a NDI^•−^ species and then by taking another electron from F^−^ it forms a NDI^2−^ dianion. When the receptor **1** is treated with TBAF a new peak at 476 nm is generated in short time irradiation and after long time irradiation a broad peak is appeared at 560 nm. In every measurement the concentration of the receptor **1** was 6 μM and TBAF concentration was 1.2 mM. Interestingly receptor **1** was not showing any chromogenic interactions with the other anions and did not show any spectroscopic changes (Figs S2 and S9).

### EPR spectroscopy

First derivative of the EPR spectra of sample solutions were recorded on a Bruker A300 spectrometer using X band. The typical experimental parameters are: microwave frequency 9.4 GHz, microwave power 0.72 mw, modulation amplitude 5 Gauss (G) at 298 K. The receptor **1** with TBAF in short time irradiation shows EPR signals but after long time irradiation the signals disappear.

### Fluorescence Measurements

Fluorescence emission spectra were recorded in a Horiba Jobin Yvon FluoroMax®-4–Spectrofluorometer. Fluorescence measurements and quenching experiments were performed on a FluoroMax-4 equipped with an injector port and stirrer at 25 °C. All experiments were performed in a quartz cell with a 1 cm path length.

### SEM imagining

The silicon wafer was cleaned by acetone, ethanol and then Milli Q water. SEM samples were prepared by solvent evaporation on a silicon wafer and then sputter coated with gold for 10 s at 0.016 mA Ar plasma (SPI, West Chester, USA) for SEM imaging using a FEI Nova NanoSEM (Hillsboro, USA) operating at high vacuum.

### Molecular modeling

Density functional theory (DFT) calculations with no consideration of dispersion interactions in gas phase were conducted using Gaussian 09 suite of programs for detail see Supplementary Information.

### ^19^F NMR spectroscopy

The recognition of fluoride ions by both the *cis* and *trans* isomers of the receptor azo-NDI was investigated by the ^19^F NMR spectroscopy with and without UV irradiation. The spectra recorded with a pure TBAF sample in the absence of the receptor displayed a broad singlet peak at −128 ppm corresponding to the F^−^ ion, and a weak doublet at −137.7 ppm corresponding to the HF_2_^−^ ion. Addition of the *trans* form of the receptor was expected to result in a slow conversion of the receptor to the corresponding anion-π sandwich with fluoride. The receptor photoisomerises to the *cis* form in the presence of UV light which forms the anion-π sandwich more easily. This was indeed observed using ^19^F NMR spectroscopy. An upfield shift of the TBAF signal was observed upon addition of the *trans* form of the receptor **1** which under the exposure of the UV light forms the **1**–fluoride sandwich rapidly. This is reflected by a small upfield shift of the fluoride resonance (ESI Fig. S4a). Further irradiation diminishes the F^−^ signal due to the electron transfer process from the F^−^ to the *cis* form of the receptor **1** forming the fluoride–NDI radical species. In the case of the *trans* isomer, in the absence of irradiation, the process was much slower (ESI Fig. S4b).

## Additional Information

**How to cite this article**: Rananaware, A. *et al*. Photomodulation of fluoride ion binding through anion-p interactions using a photoswitchable azobenzene system. *Sci. Rep.* **6**, 22928; doi: 10.1038/srep22928 (2016).

## Supplementary Material

Supplementary Information
