# Supplementary material for: Photomodulation of fluoride ion binding through anion-π interactions using a photoswitchable azobenzene system
Source: Sci Rep. 2016 Mar 8;6:22928. doi: 10.1038/srep22928 (PMC4782172; doi:10.1038/srep22928)
Supplement: Supplementary Information [file srep22928-s1.pdf]

Supplementary Information

Photomodulation of fluoride ion binding through anion-π interactions using a photoswitchable azobenzene system

Anushri Rananaware,[1,†] Mousumi Samanta,[2,†] Rajesh S. Bhosale,[3] Mohammad Al Kobaisi,[1] Biswajit Roy,[2] Varun Bheemireddy,[2] Sidhanath V. Bhosale,[3] Subhajit Bandyopadhyay,[2]* Sheshanath V. Bhosale[1*]

[1]School of Applied Sciences, RMIT University, GPO Box 2476, Melbourne, VIC-3001, Australia. [2]Department of Chemical Sciences, Indian Institute of Science Education and Research Kolkata, Mohanpur, Nadia WB 741246 India, [3]Polymers and Functional Materials Division, CSIR-Indian Institute of Chemical Technology, Hyderabad, Telangana-500007, India. [†] These authors contributed equally.

Electrochemical experimental method

Electrochemistry was undertaken in freshly dry DMF (over calcium hydride), with 0.1 M tetrabutyl ammonium hexafluorophosphate ($TBAPF_6$) as a supporting electrolyte, which was purchased from Aldrich (Electrochemical grade) and twice distilled from EtOH before use. A Glassy carbon (GC) electrode was used as a working electrode (ALS, Japan), which was polished with 0.05 µm alumina on a felt pad, washed with distilled water then EtOH and dried under a N_2 stream before use. A platinum wire was used as a counter electrode and a silver wire was used as a pseudo reference electrode. All experiments were undertaken in N_2 purged solvent, with a N_2 blanket maintained over the solution

during the experiments. Ferrocene was used as an internal reference by doping all solutions with an approximately equimolar amount of ferrocene, and all potentials are referred to this internal standard as recommended by IUPAC Voltammograms displayed in the paper were recorded with a scan rate of 50 mV/s.[S1] Increasing scan rates to 500 mV/s had minimal effect on peak potentials for reversible processes and no change in $E_{1/2}$ values. $E_{1/2}$ values are taken as the half-way point between the forward and reverse peak for each reversible redox process. $E_{1/2}$ potentials were confirmed by Differential Pulse Voltammetry (DPV) to resolve overlapping peaks and minimise background current.

The cyclic voltammetry results of **1** displayed two well resolved, reversible one-electron reduction waves corresponding to the formation of the $NDI^{\bullet-}$ and NDI^{2-} species, respectively (Fig. 4d in main manuscript).

Electrospray Ionisation Mass Spectrometry (ESI-MS)

ESI-MS were recorded on an Acquity™ ultra performance LC using ESI-MS recorded in the negative-ion mode ionisation mode for the detection of the complexes of receptor **1** and F^- ion. The recorded mass spectra are showing in Figure S5.

Synthesis of azo-NDI (1)

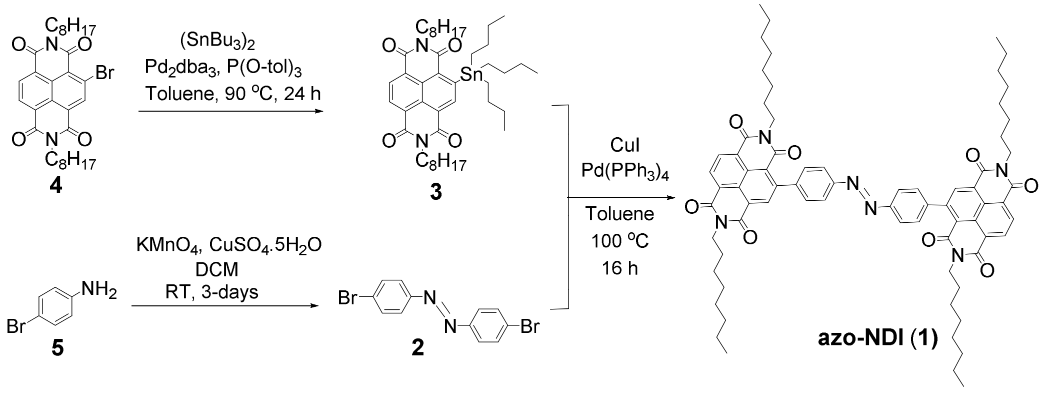

To a stirred solution of **2** (0.072 g, 0.2130 mmol) in anhydrous toluene (15 mL) was added CuI (cat.), **3** (0.5 g, 0.641 mmol) and resultant solution was stirred at RT for 15 min under argon atmosphere. Then $Pd(PPh_3)_4$ (0.025 g, 0.0022 mmol) was added and reaction mixture was refluxed for 16 h. Reaction completion was checked by TLC analysis. After completion, solvent was evaporated under reduced pressure and the obtained crude residue was purified by flash column chromatography to give **5** (0.15 g, 61%) as an orange coloured solid. ^{1}H NMR (300 MHz, $CDCl_3$): δ 8.76 (dd, J = 18.4, 7.7 Hz, 4H), 8.57 (s, 2H), 8.05 (d, J = 8.5 Hz, 4H), 7.50 (d, J = 8.4 Hz, 4H), 4.19–3.98 (m, 8H), 1.77–1.51 (m, 8H), 1.44–1.07 (m, 40H), 0.88–0.72 (m, 12H); ^{13}C NMR (300 MHz, $CDCl_3$): δ 162.86, 162.62, 162.23, 152.25, 147.25, 143.38, 135.08, 131.46, 130.72, 129.02, 127.77, 126.98, 126.67, 126.27, 125.48, 123.15, 41.13, 41.03, 31.80, 31.77, 29.29, 29.21, 29.19, 28.09, 27.08, 22.63, 14.08; HRMS (MALDI-TOF, m/z): [M^+] calcd for $C_{72}H_{82}N_6O_8$: 1158.62, found: 1159.50 $[M+H]^+$, 1181.48 $[M+Na]^+$.

Synthesis of intermediate 2 and 3

Synthesis of 1,2-bis(4-bromophenyl)diazene (**2**).[S2]

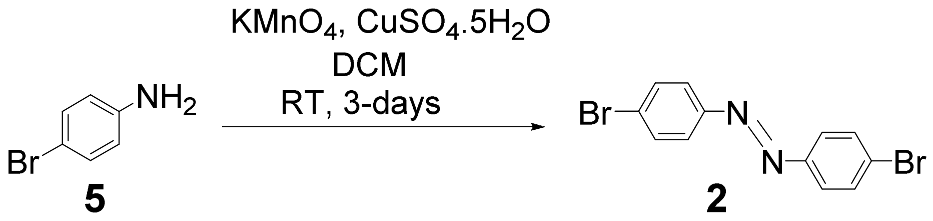

A finely grinded mixture of $KMnO_4$ (2.9 g, 18.38 mmol) and $CuSO_4.5H_2O$ (2.9 g, 18.38 mmol) was added to a stirred solution of 4-bromo aniline **5** (1 g, 13.7 mmol) in degassed DCM (80 mL) and resultant mixture was stirred at room temperature for 3 days. The completion of reaction was monitored by TLC analysis. After completion, reaction mixture was filtered through celite bed and the residue washed with excess of DCM. Filtrate was evaporated to dryness and the obtained crude product was purified by column chromatography to afford **2** (1.8 g, 91 %) as an orange solid. ^{1}H NMR (300 MHz, $CDCl_3$): δ 7.89 (d, *J* = 8.52 Hz, 4H), 7.66 (d, *J* = 8.8 Hz, 4H); ^{13}C NMR (300 MHz, $CDCl_3$): δ 151.17, 132.42, 125.77, 124.43; HR-MS (EI) calcd for $C_{12}H_8Br_2N_2$ ($[M]^+$), 337.91; found, 340.90.

Synthesis of 2,7-dioctyl-4-(tributylstannyl)benzo[lmn][3,8] phenanthroline-1,3,6,8(2H,7H)-tetraone **3**.[S3]

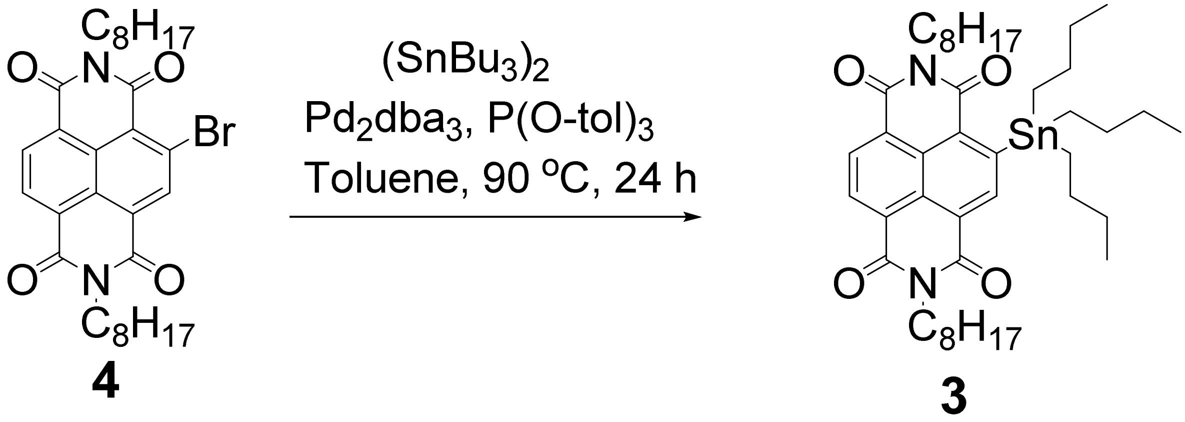

This compound was prepared following known literature method. Typically, to a stirred solution of **4** (2.0 g, 3.519 mmol) in dry toluene (50 mL) was added hexabutylditin (2.04 g, 3.519 mmol), P(o-tol)$_3$ (0.214 g, 0.2 mmol) and was degassed for 10 min using nitrogen. Then to this $Pd_2(dba)_3$ (0.161 g, 0.05 mmol) was added and resulting reaction mixture was refluxed for 24h. The completion of reaction was monitored by TLC analysis. After completion, reaction mixture was cooled to room temperature, the solvent was concentrated under reduced pressure and obtained crude residue was further purified by silica gel chromatography affording **3** (1.98 g, 72%) as an orange-yellow solid. Characterisation of **3** perfectly matches with literature values. ^{1}H NMR (300 MHz $CDCl_3$): δ 8.98 (s, 1H), 8.72–8.71 (m, 2 H), 4.21–4.19 (m, 4H), 1.74–1.73 (m, 4H), 1.55–1.20 (m, 38H), 0.89–0.86 (m, 15H).

Density Functional Theory (DFT) calculations

Gaussian 09 package was used for all the theoretical calculations.[S4] The geometry optimisation of *cis*-**azo-NDI** (**2**) was performed in the aqueous phase using Density Functional Theory(DFT) with BLYP-D functional [S5,S6] and aug-cc-pvdz basis set.S7 In order to evaluate the effect of solvent on the geometry and energetics, conductor-like polarizable continuum model (CPCM)[S8,S9] of nitromethane ($\varepsilon_r = 35.8$) was implemented.

The energy minimised structure of the *cis* isomer of azo NDI **1** was obtained from theoretical calculations by using Gaussian 09 package. In the *cis* form, the two NDI units are nearly stacked and providing a suitable conformation for anion–π interaction when F□ is sandwiched between the two NDI moieties.

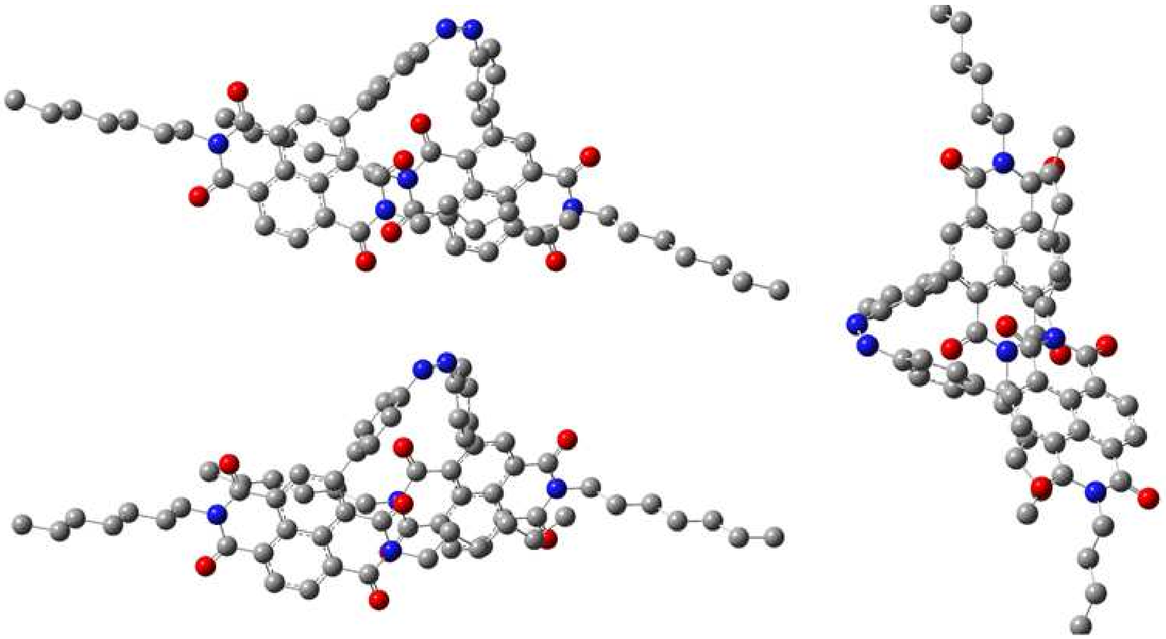

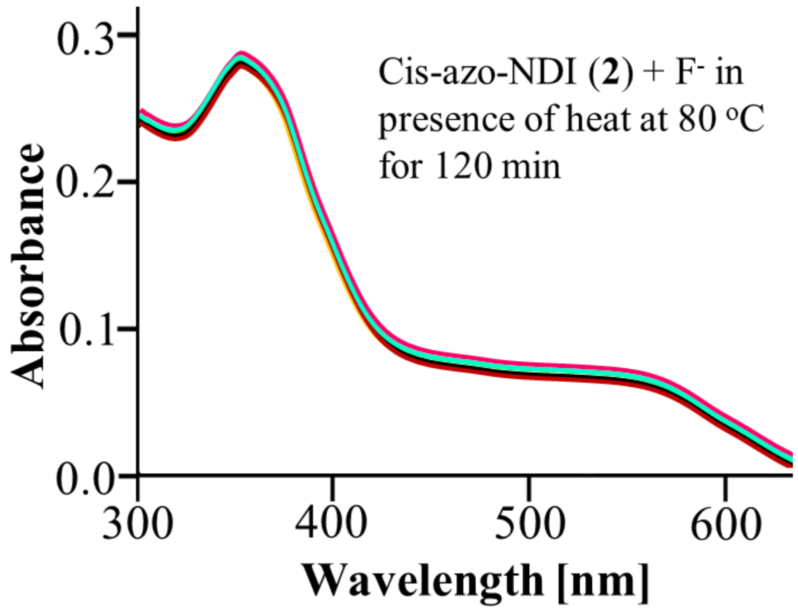

Figure S1 | Attempted conversion of *cis*-to-*trans* isomerisation in DMF under thermal conditions. The spectra indicate that the changes were insignificant.

To test our hypothesis, we employed azo-NDI (**1**) with various anions such as F^-, Cl^-, Br^-, I^-, AcO^-, and $H_2PO_4^-$ anions in their tetra-*n*-butylammonium (TBA) salts with and without UV light irradiation (λ_{ex} = 366 nm). Despite anions being in excess (200 equiv.) only F^- showed form NDI/F^-/NDI sandwich complex (ESI Fig. S2). These results clearly suggest that there are no electronic interactions of the **azo-NDI** (**1**) with larger anions compared to fluoride.

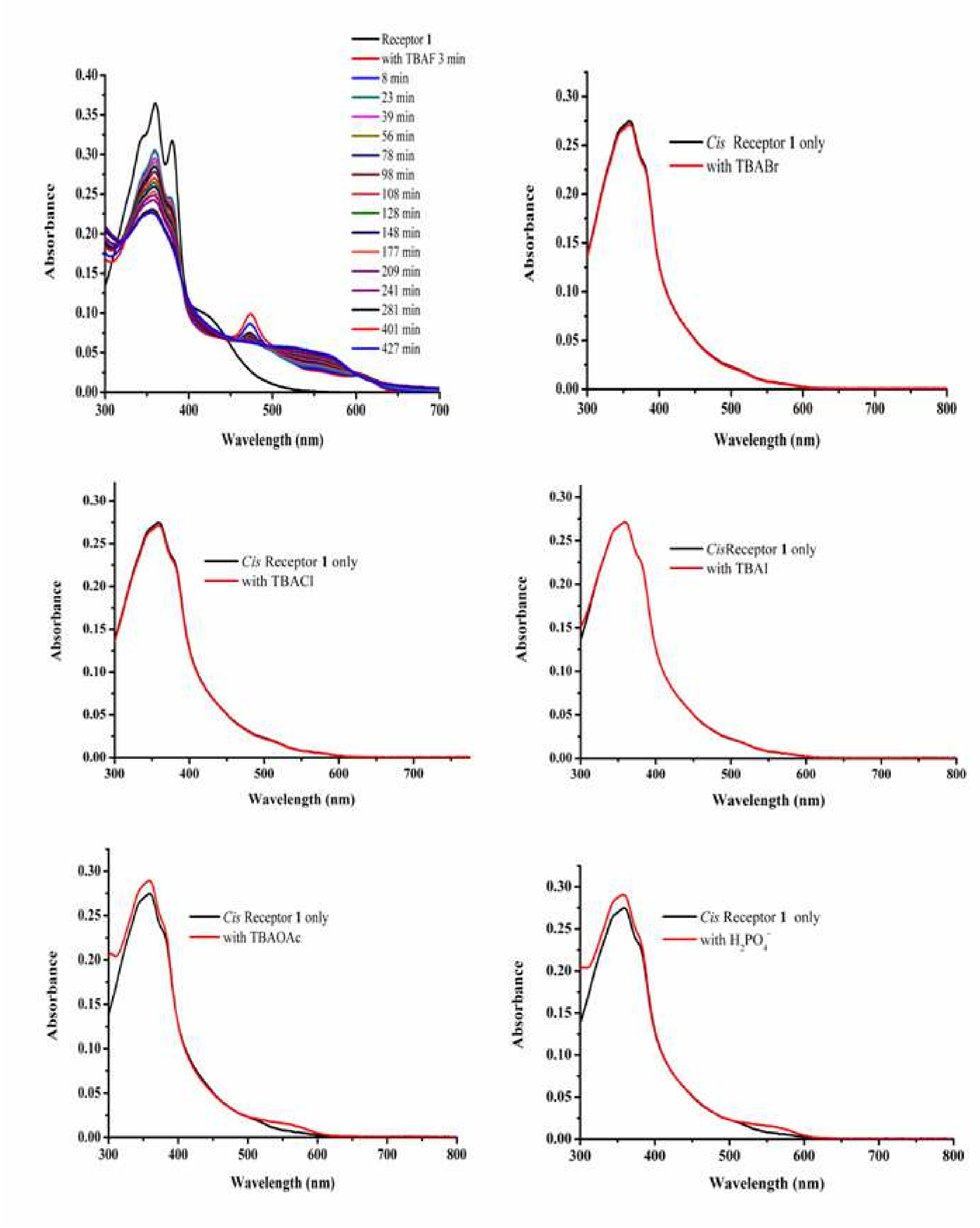

Figure S2 | UV-vis spectra of receptor **1** (6 µM) with UV irradiation at 366 nm in the presence of TBAX (1.2 mM) (X = F-, Cl^-, I^-, Br^-,OAc^-, $H_2PO_4^-$) in DMF.

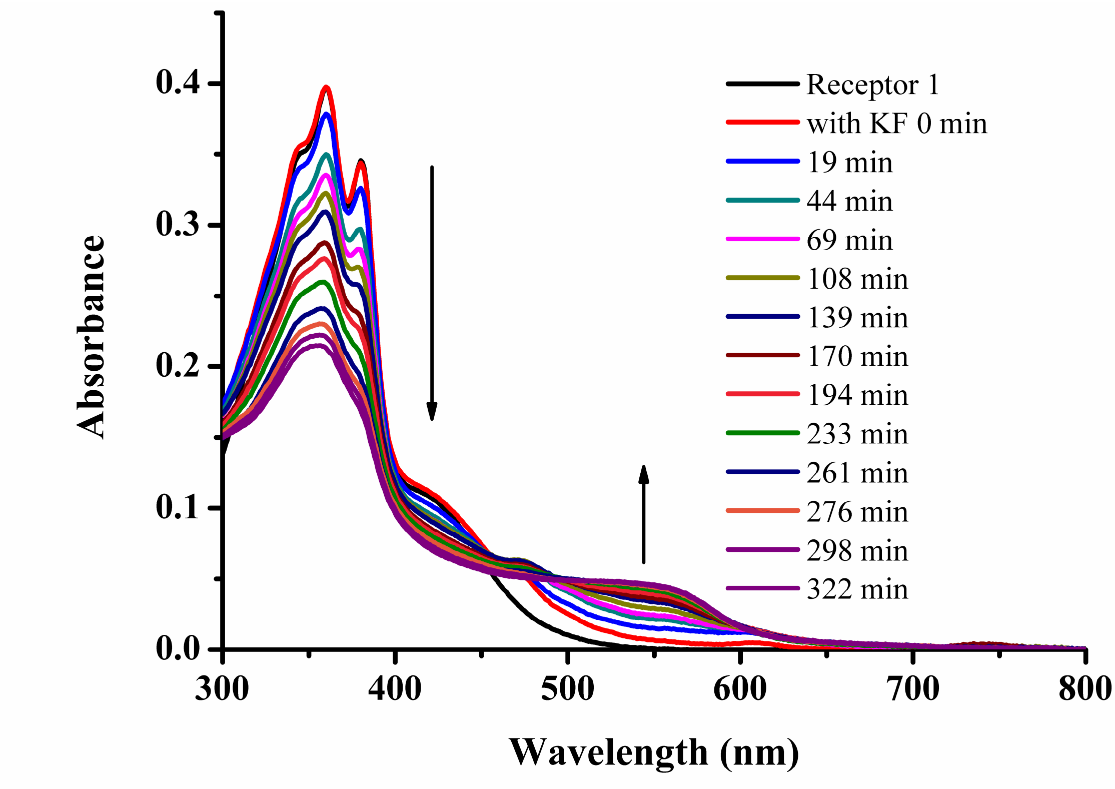

Figure S3 | UV-vis spectra of receptor **1** (6 µM) with KF (1.2 mM) in DMF in presence of 366 nm light, which also confirms formation of $NDI^{\bullet -}$ and NDI^{2-} stable species and independent of fluoride source.

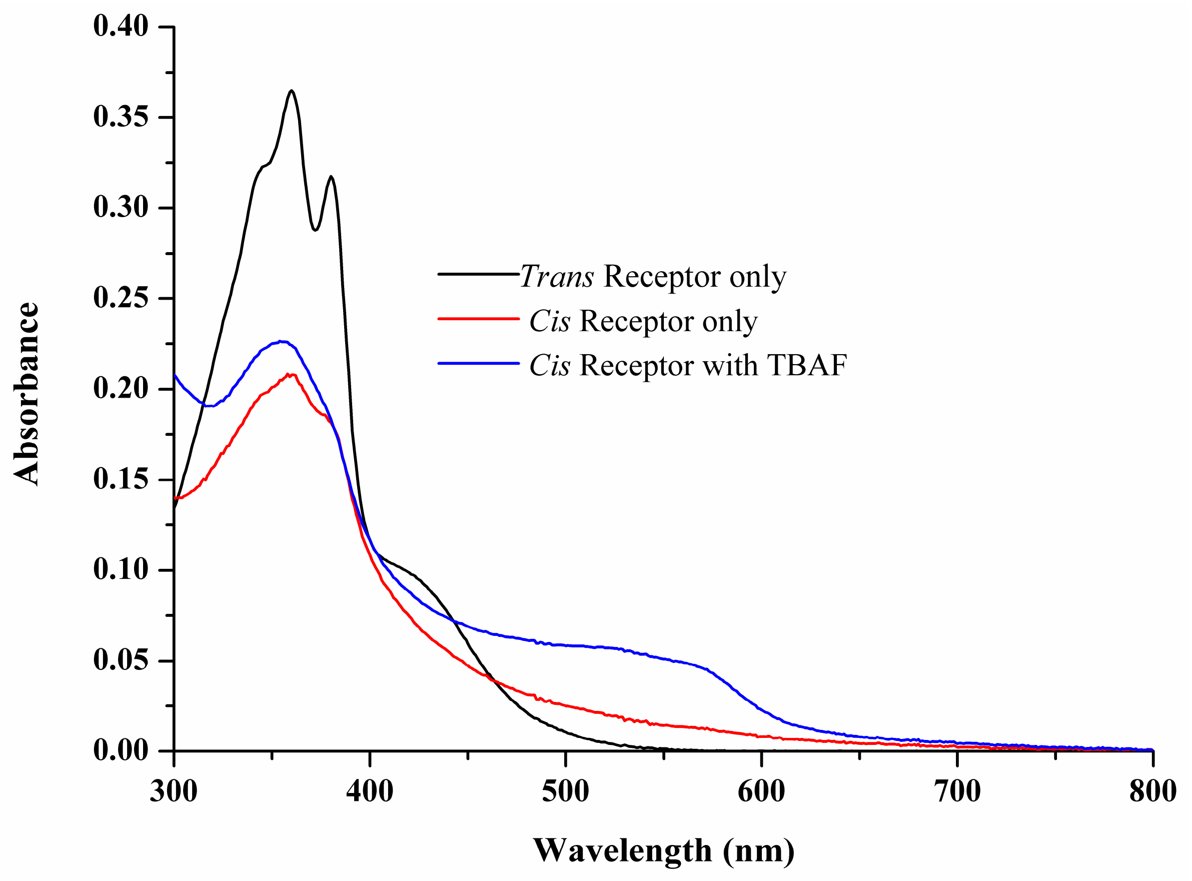

Figure S4 | UV-vis spectra of receptor 6 μM in DMF: **a**, *trans*-**azo-NDI** only, **b**, upon irradiation with UV light at 366 nm, which confirms formation of *cis*-**azo-NDI** and **c**, the peak of *cis*-**azo-NDI** upon addition of 20 equiv. of F^- (TBA salt). These results clearly differentiate difference between *cis*-**azo-NDI** and formation of NDI radical and dianions in the presence of F^- anions, respectively

^{19}F NMR spectroscopy

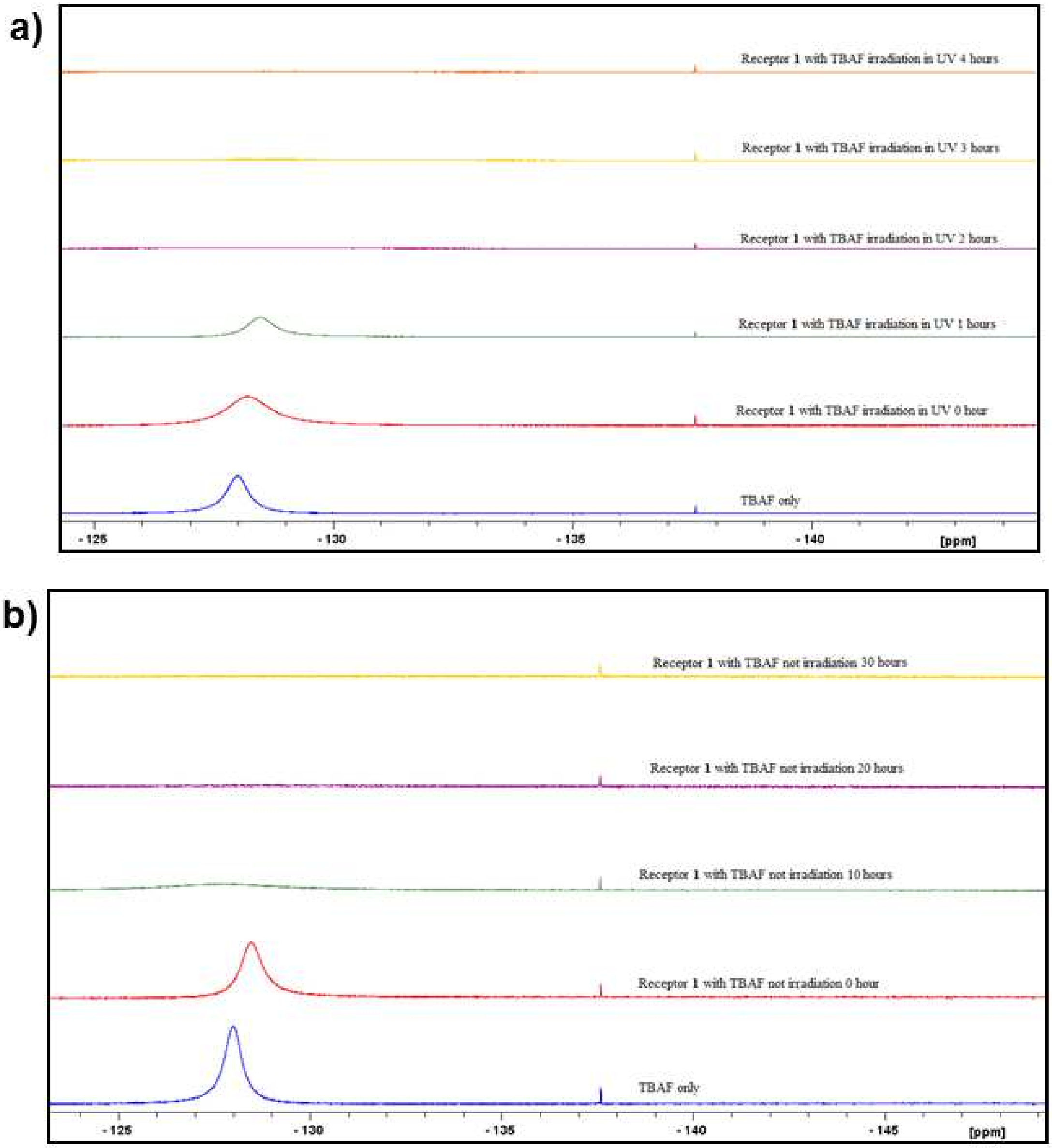

Figure S5 | ^{19}F NMR spectra of the receptor **1** with TBAF, **a**, in presence of UV light of 366 nm (Acetone-d_6) and; **b**, without irradiation (Acetone-d_6).

The formation of NDI/F^-/NDI sandwich dianion was also evident from the electrospray ionization mass spectrometry (ESI-MS) recorded in the negative-ion mode. Although, it is well recognized that the negative-ion mode is less sensitive compared to the positive ion mode, clear peaks at 580 and 602 were observed that correspond to the mass of the NDI^{2-} of *cis*-**azo-NDI** (**2**) receptor associated with H^+ and Na^+ ions, respectively (ESI Fig S5). No peak of the fluoride-receptor anion complex was obtained in the mass spectrometry, perhaps because of the lack of the stability of such species in the gas state under which the mass spectrometry experiments are performed. These results confirm that the formation of covalent C-F bond rule out the possibilities. This clearly suggest that NDI/F^-/NDI complex facilitate the F^-→NDI electron transfer that generated the NDI anions.

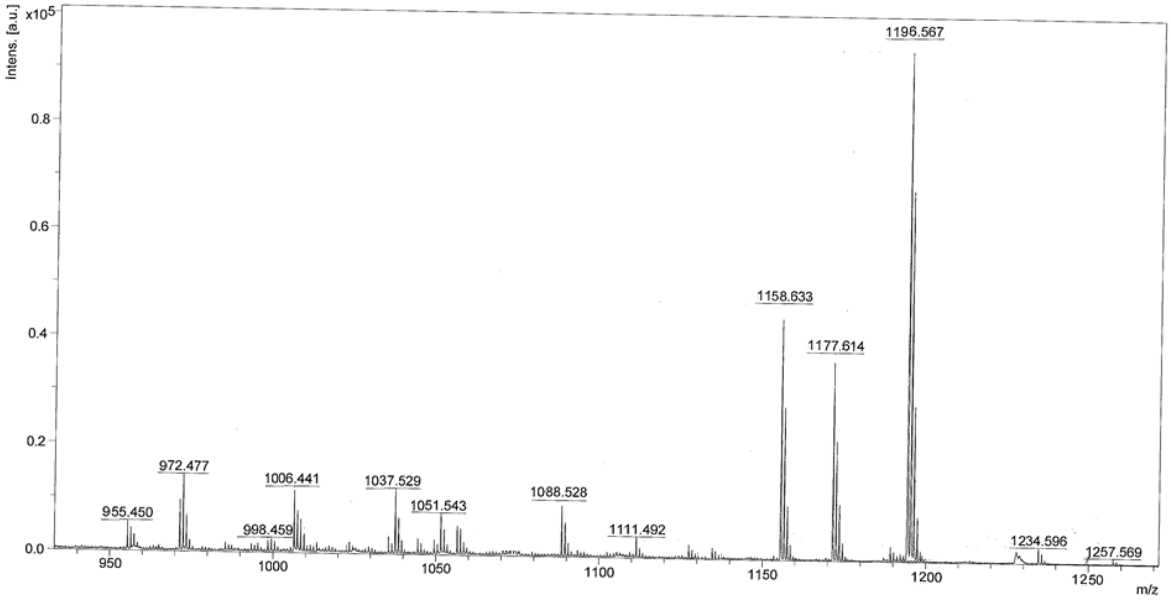

Figure S6 | ESI-MS spectra *cis*-**azo-NDI** in the presence of F^- ion.

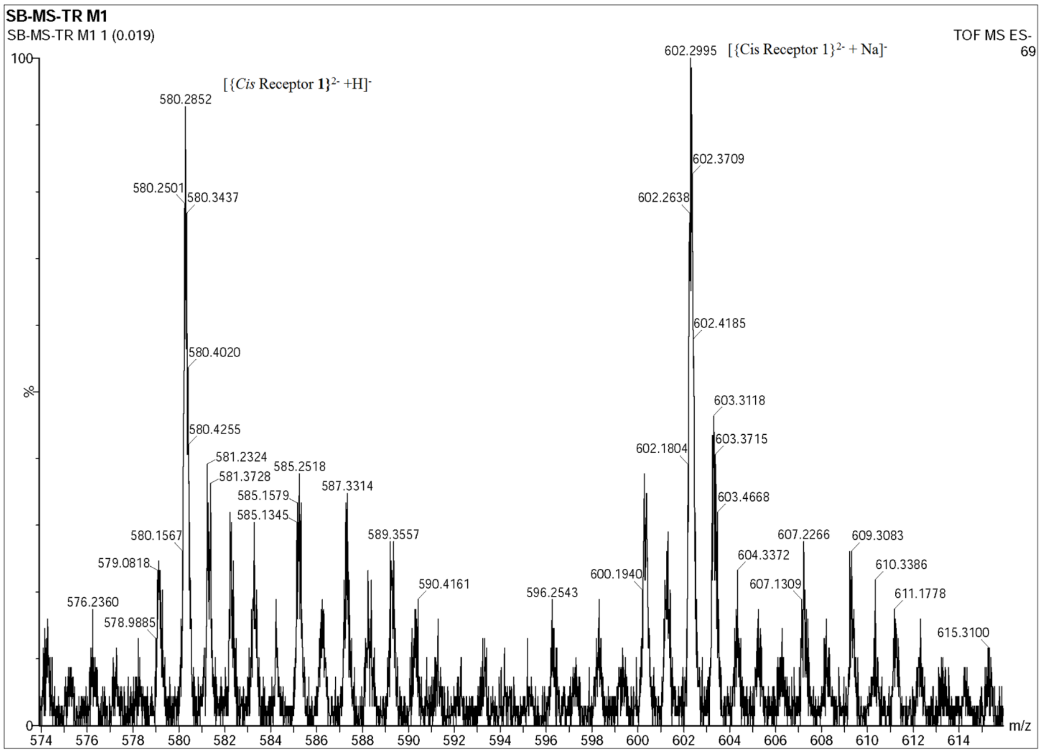

Figure S7 | ESI-MS spectra showing NDI^{2-} ion.

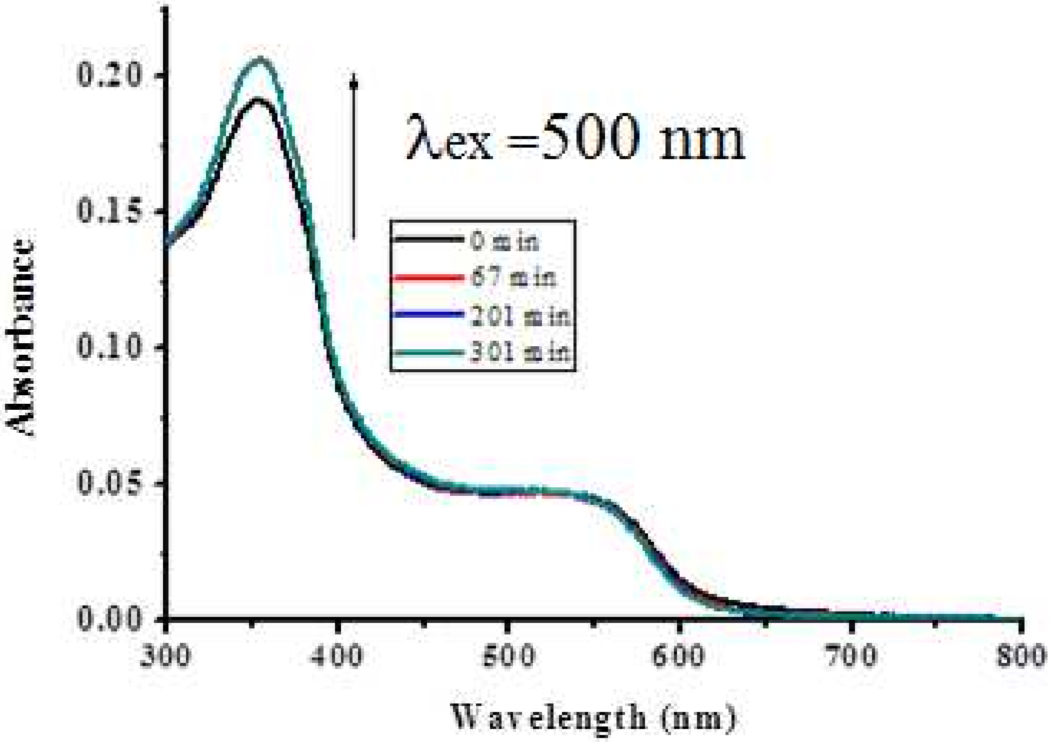

Figure S8 | UV-vis spectra of receptor **1** (6 μM) with F$^-$ (1.2 mM) in DMF in presence of visible light (500 nm), no *cis* to *trans* isomerisation was observed.

It should be noted that the *trans* form of the receptor azo-NDI **1**, however weakly interaction of NDI with F^- as illustrated in ESI Figure S7. Typically, NDI-F^- interaction takes place in about 400 min without UV irradiation, however in the presence of UV (366 nm) light it takes about 100 min., which can be seen by colour changes and UV-vis absorption spectroscopy. The fluorescence spectra also confirm these interactions. Importantly, upon oxidation of NDI radicals gives original **azo-NDI** absorption spectrum and also shown original pale yellow color of **1**. The **azo-NDI** (**1**) does not have any interactions with other anions (Figure S8).

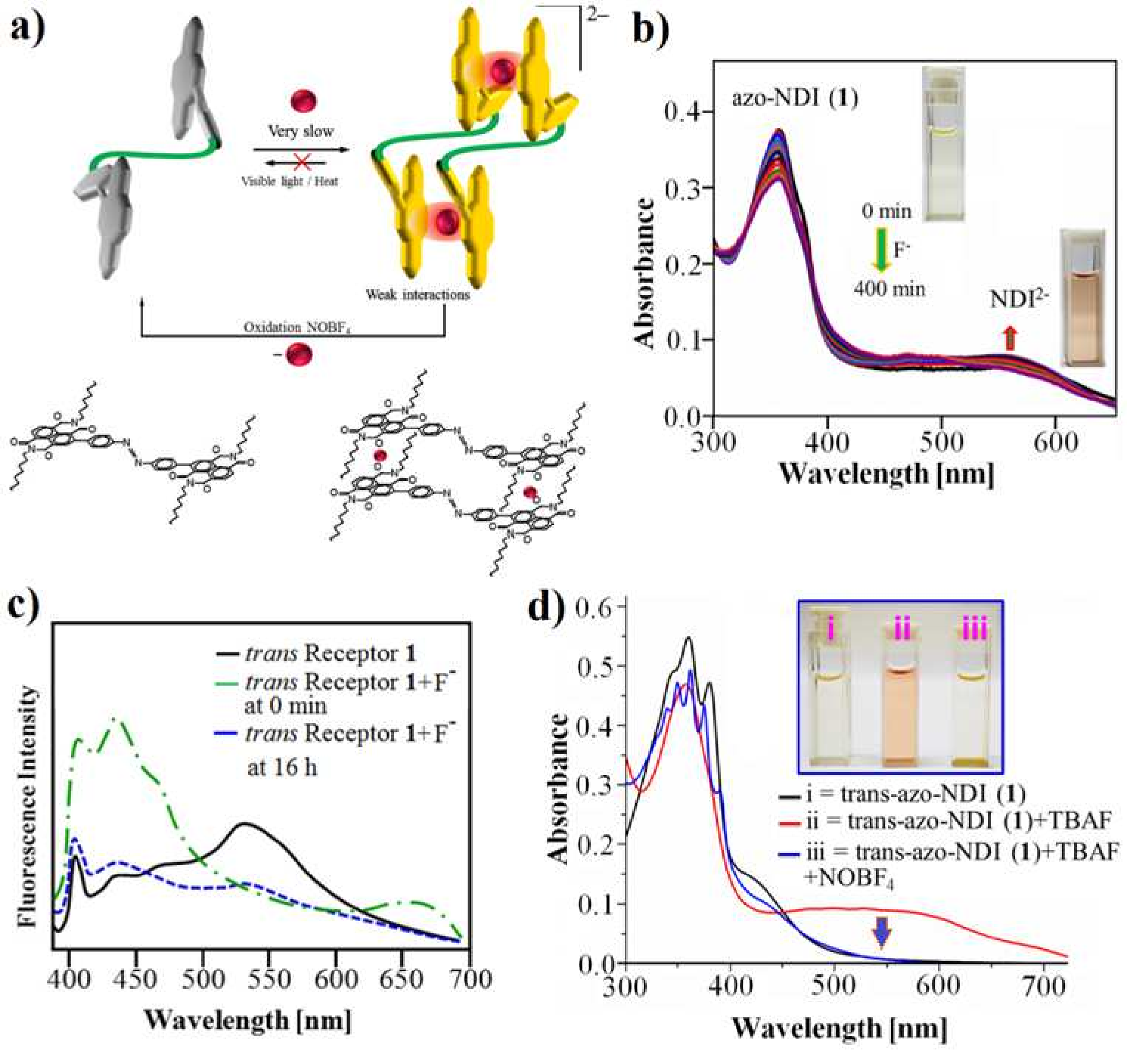

Figure S9 | UV-vis spectra of receptor **1** (6 µM) with 1.2 mM $TBAF^-$ in DMF without irradiation of UV light.

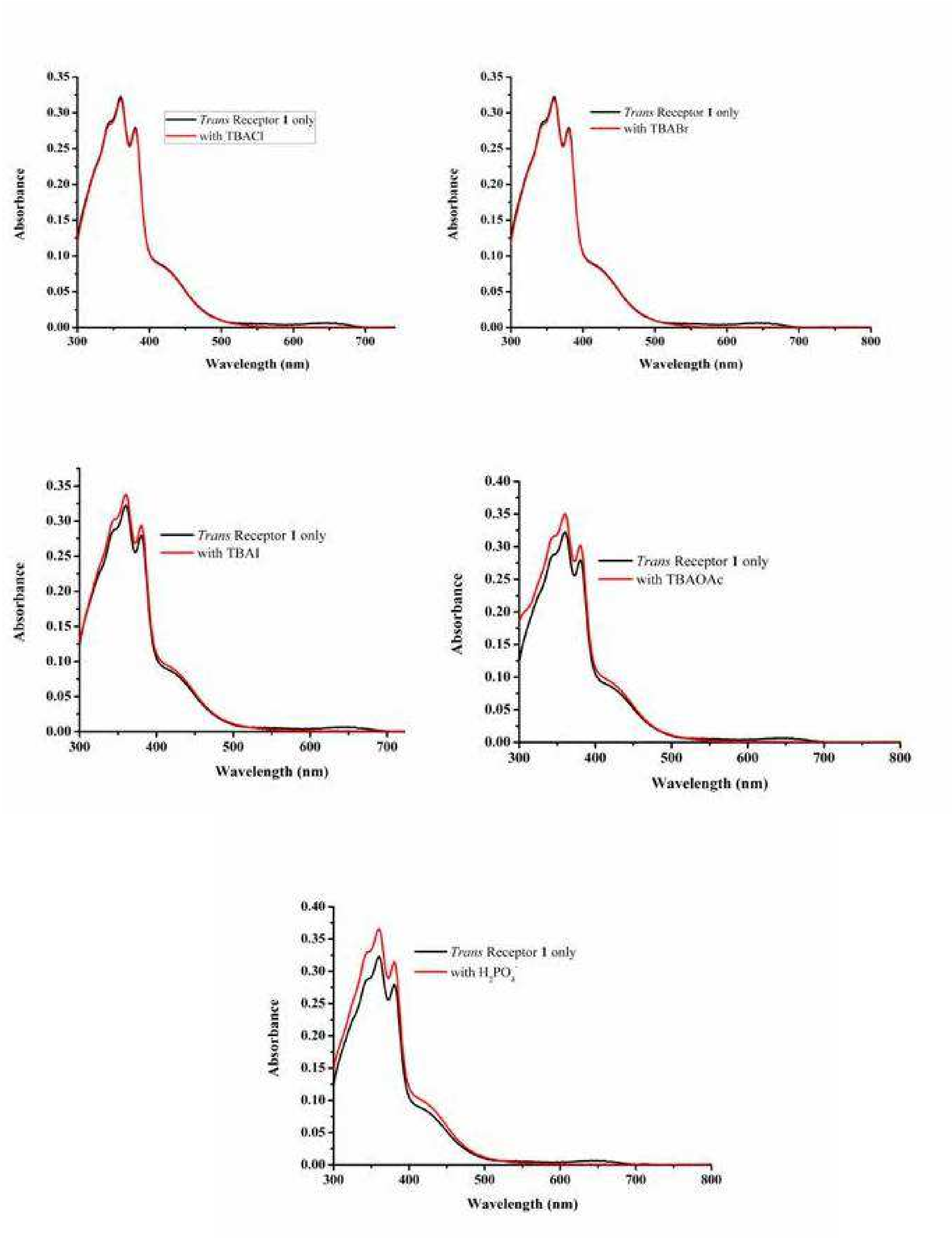

Figure S10 | UV-vis spectra of receptor **1** in *trans* form (6 µM) with 1.2 mM TBAX (X = Cl^-, I^-, Br^-,OAc^-, $H_2PO_4^-$ in DMF without irradiation of UV light.

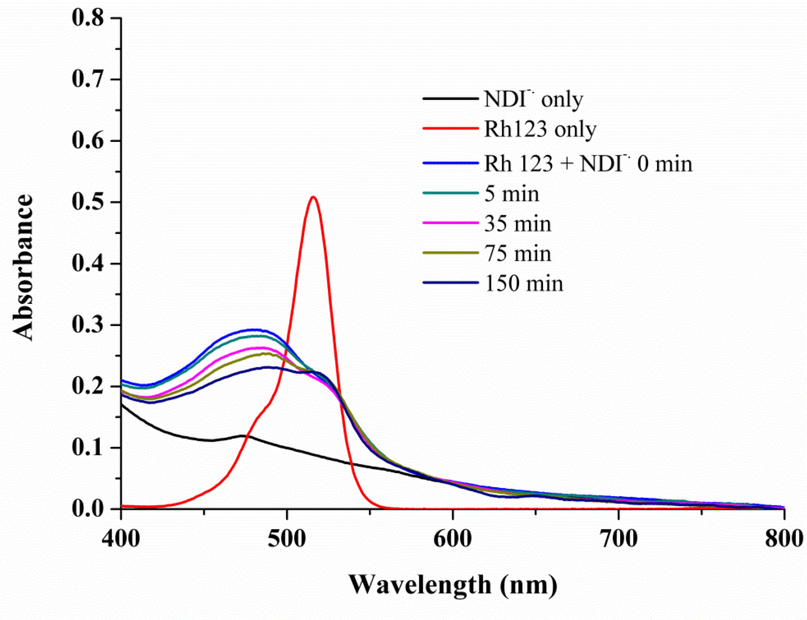

Figure S11 | UV-vis spectra of receptor of the degradation of Rh-123 by the NDI$^{•-}$ stable radical anion obtained from the *cis* form of the switch over the time.

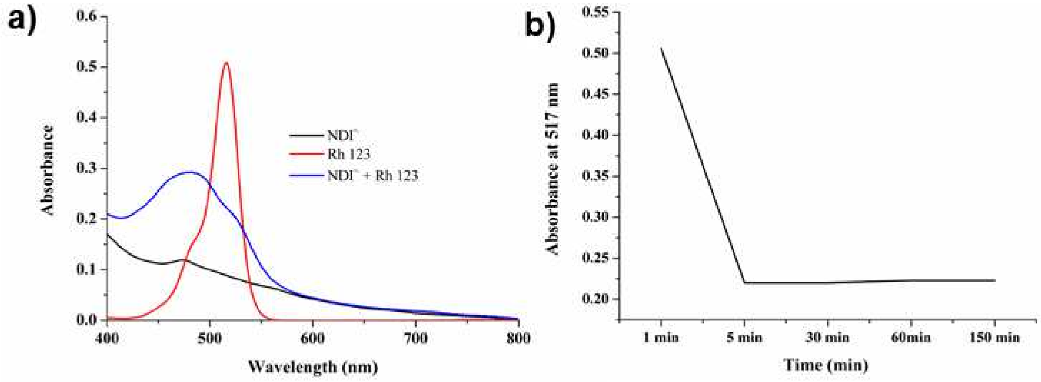

Figure S12 | The degradation of Rh-123 by the NDI$^{•-}$ stable radical anion obtained from the *cis* form of the switch. a, The absorption spectra of Rh123 in the presence of NDI$^{•-}$ anions within 5 min. **b**, Changes in absorbance values of Rh 123 monitored at 517 nm at various time intervals in the presence of the NDI radical ion.

Dynamic light scattering (DLS)

DLS measurements were performed using a Malvern Nano-ZS zetasizer (Malvern Instruments Ltd, Worcestershire, United Kingdom). The sample is illuminated by a 633 nm He–Ne laser and a maximum output power of 4 mW power was used as light source. Measurements were performed at scattered light is measured at a 173° accumulation angle. The size distribution of the aggregates was calculated from the diffusion coefficient of the particles according to Stokes-Einstein equation. The average diameter and the polydispersity index of the samples are calculated by the software using CONTIN analysis. Freshly prepared samples of **1** or **2** with a concentration of 1x10^{-4} M in DMF were used for DLS measurements.

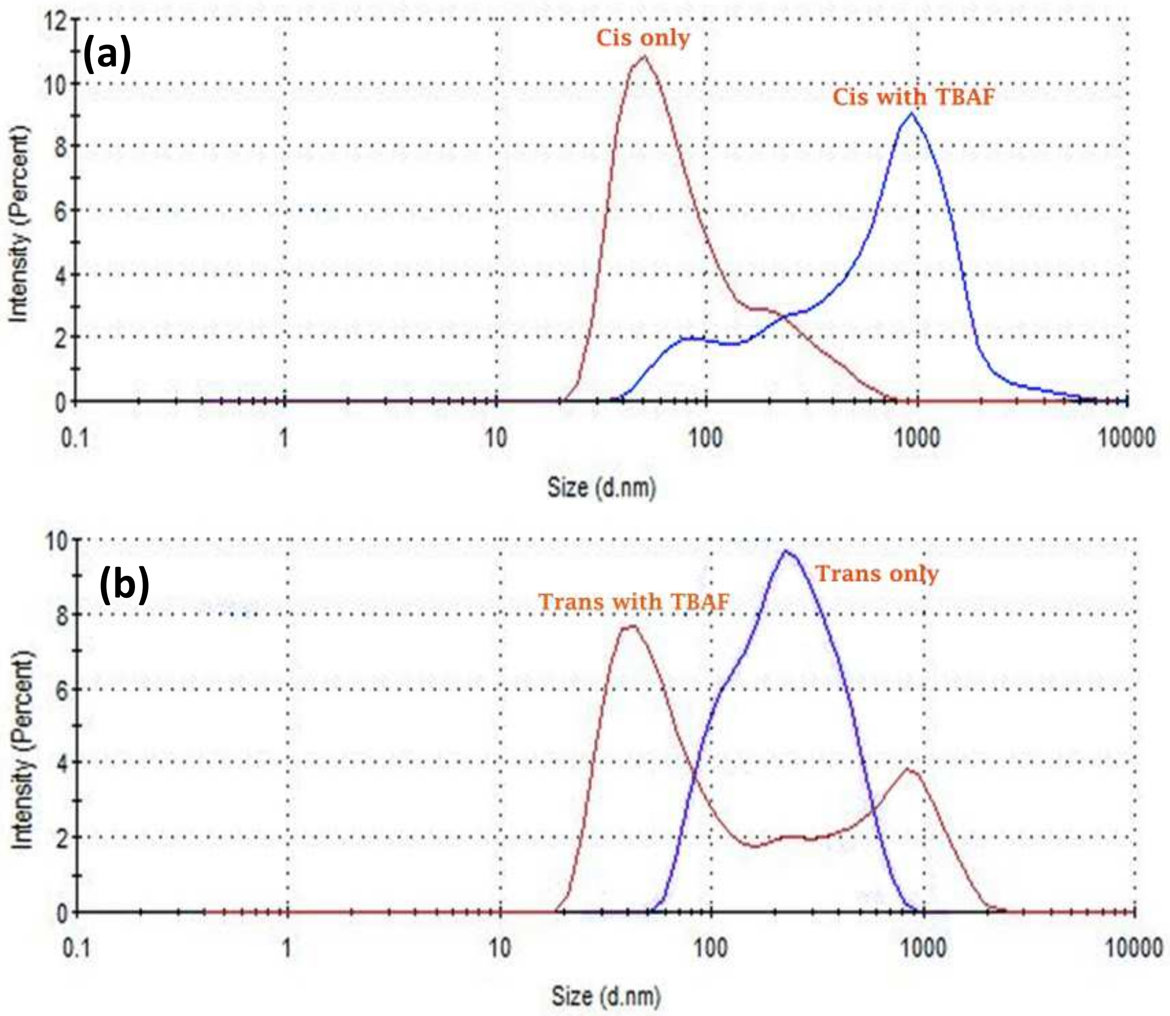

Figure S13 | The hydrodynamic diameter distribution of: **a**,*cis*, and **b**, *trans* isomers of **azo-NDI** in their molecular and salt forms as measured using dynamic light scattering (DLS) particle size analyser.

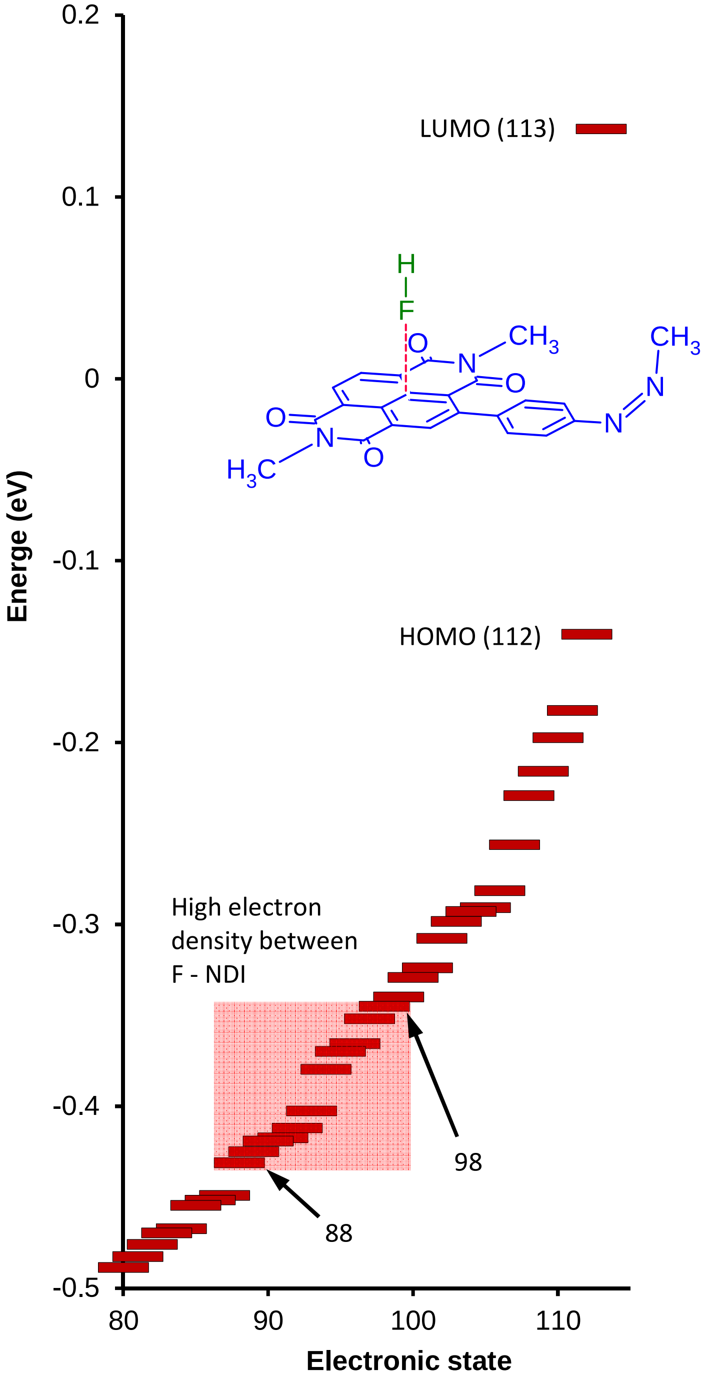

Figure S14 | The electronic energy states of the HF–azo-NDI model as calculated using Hartree-Fock and 3-21g.

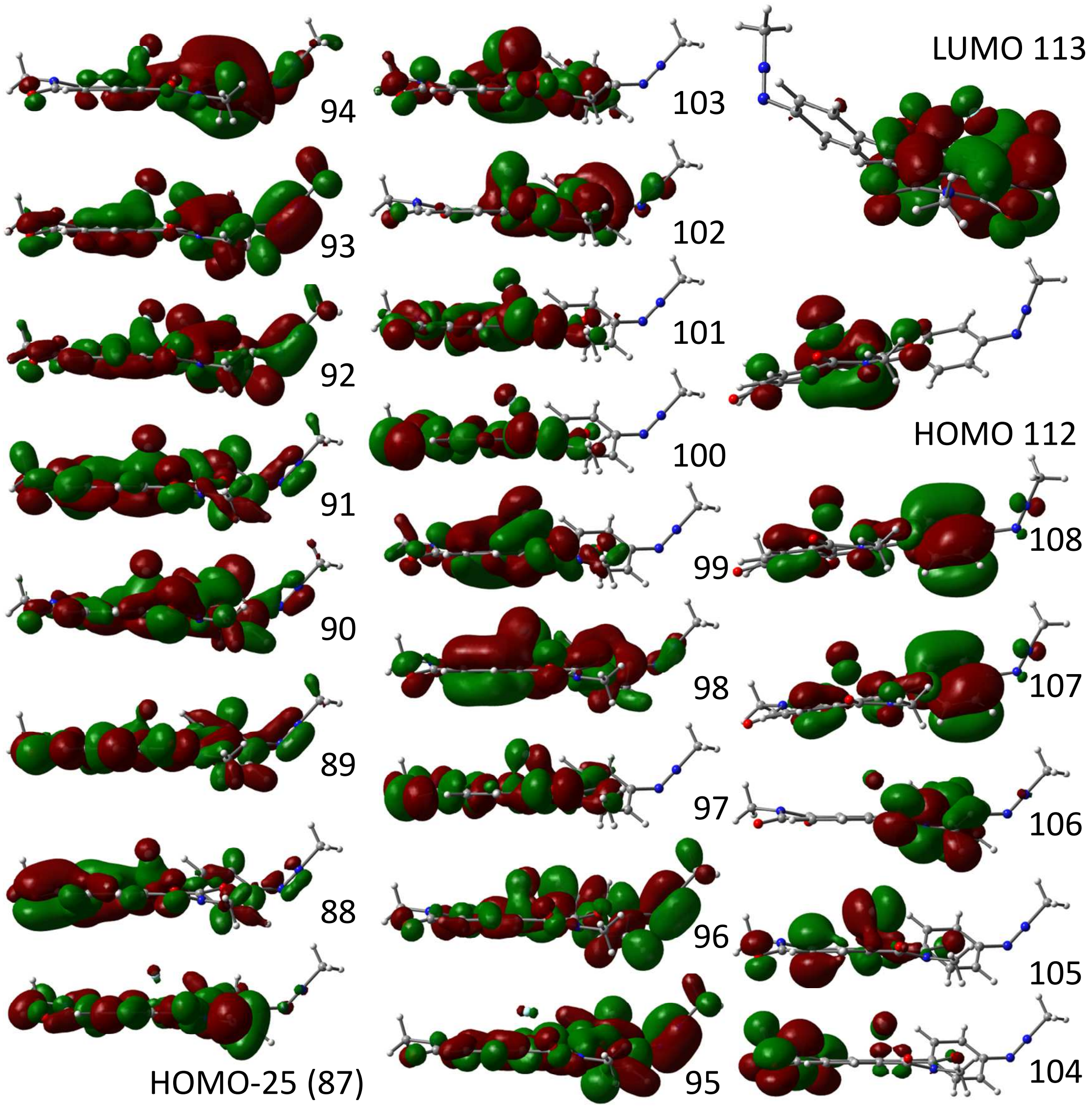

Figure S15 | Orbital density distribution for molecular orbitals from HOMO-25 to HOMO-4 and HOMO and LUMO of HF–**azo-NDI** model calculated using Hartree- Fock and 3-21g level of theory.

Spectroscopic characterisation (^{1}H-NMR, ^{13}C-NMR and Mass spectroscopy)

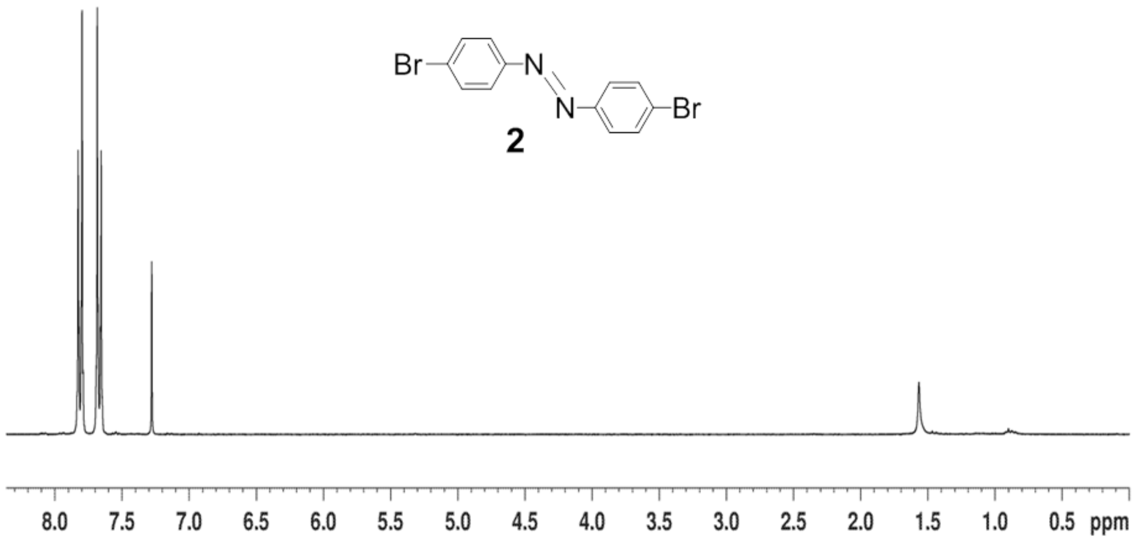

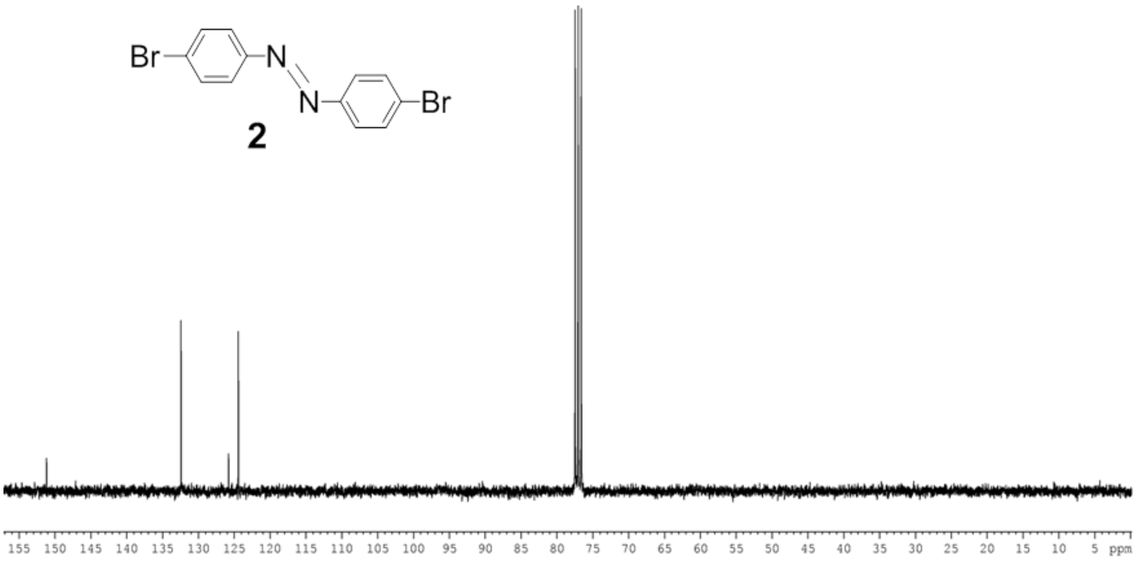

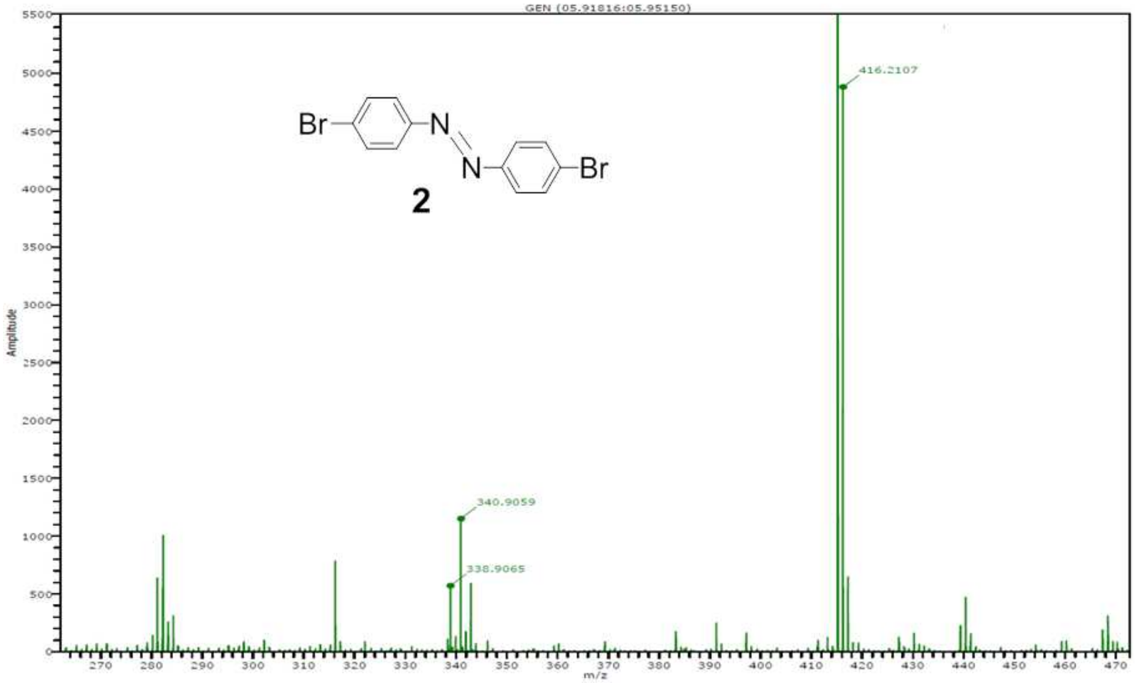
GEN (05.91816:05.95150)
416.2107
340.9059
338.9065
Br
N
N
Br
2
Amplitude
m/z

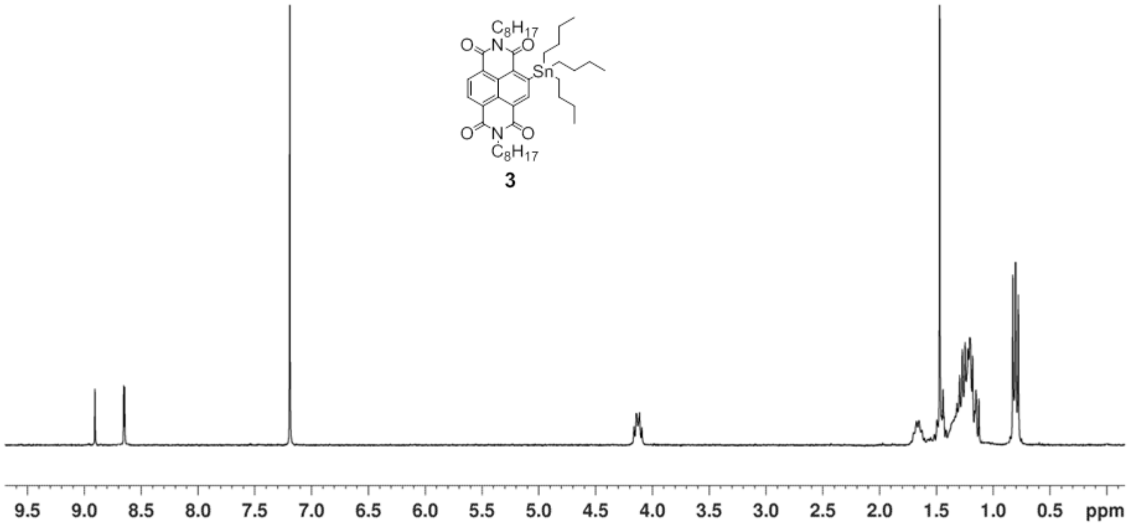
C8H17
O
N
O
Sn
O
N
O
C8H17
3
9.5 9.0 8.5 8.0 7.5 7.0 6.5 6.0 5.5 5.0 4.5 4.0 3.5 3.0 2.5 2.0 1.5 1.0 0.5 ppm

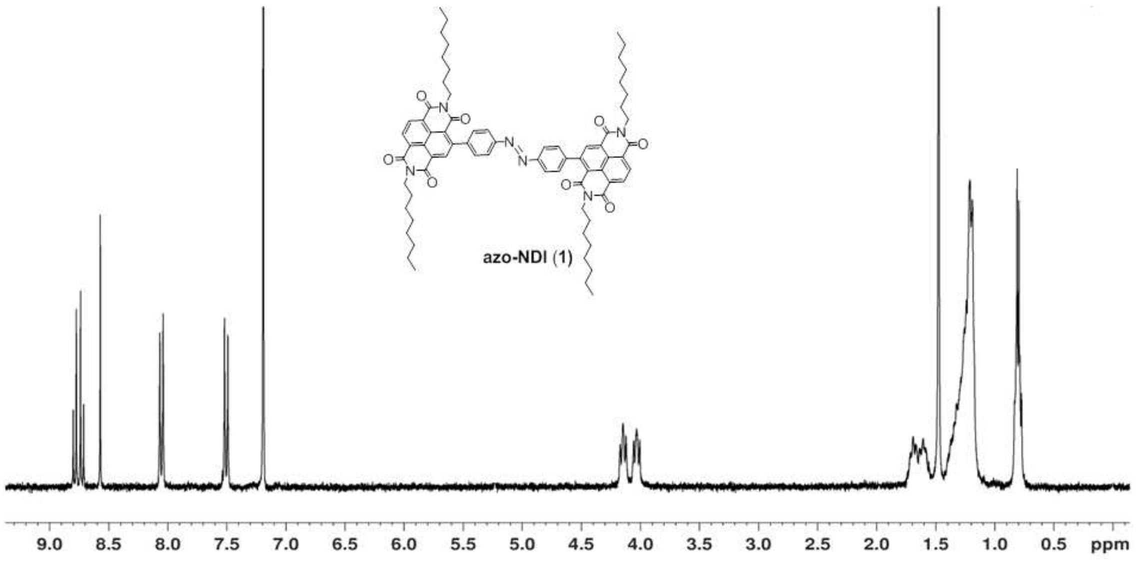
azo-NDI (1)
9.0 8.5 8.0 7.5 7.0 6.5 6.0 5.5 5.0 4.5 4.0 3.5 3.0 2.5 2.0 1.5 1.0 0.5 ppm

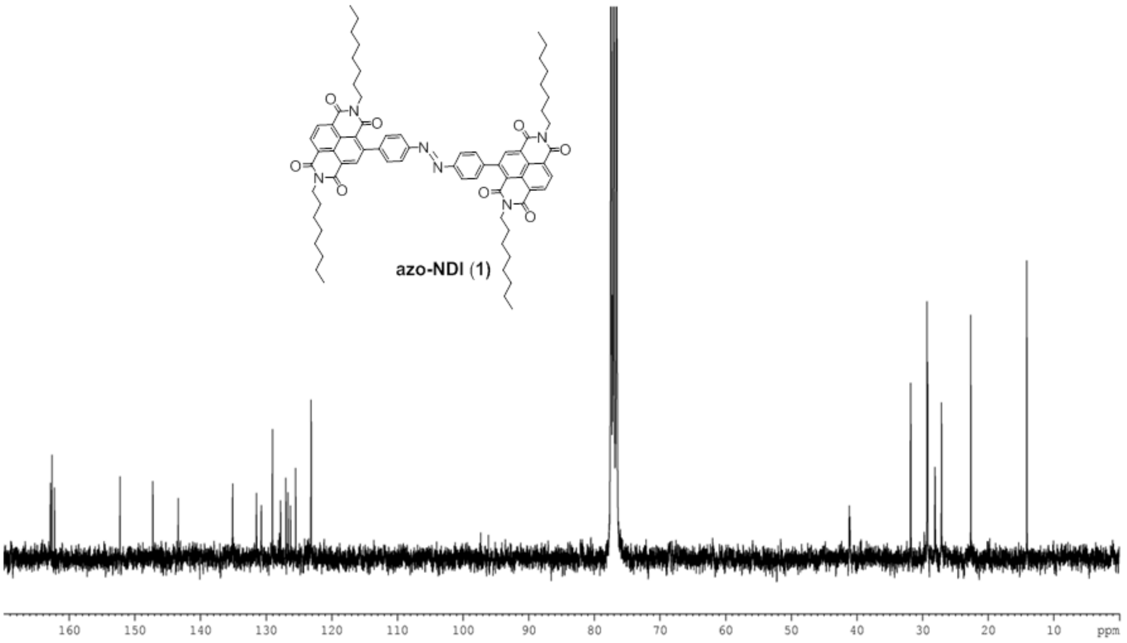
azo-NDI (1)
160 150 140 130 120 110 100 90 80 70 60 50 40 30 20 10 ppm

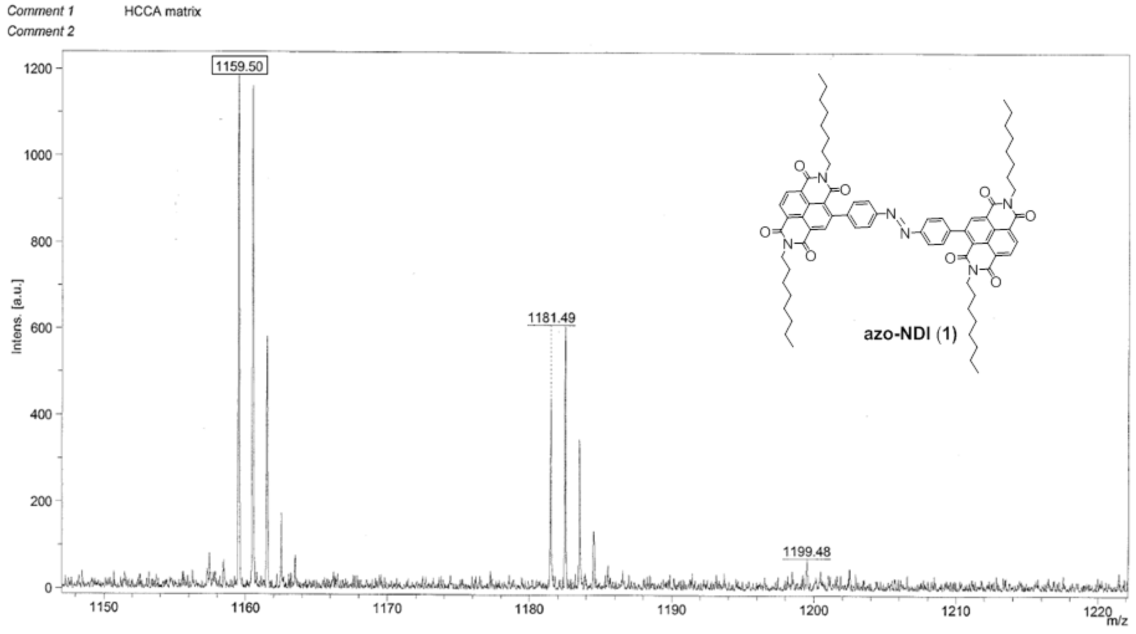

References

S1. Gritzner, G. Polarographic half-wave potentials of cations in nonaqueous solventsPure and Applied Chemistry **1990**; *62*, 1839-58.

S2 Grebel-Koehler, D. *et al.* Synthesis and Photomodulation of Rigid Polyphenylene Dendrimers with an Azobenzene Core *Macromolecules*, **2003**, *36*, 578–590.

S3 Polander, Lauren E. Stannyl Derivatives of Naphthalene Diimides and Their Use in Oligomer Synthesis *Org. Lett.*, **2012**, *14*, 918-921.

S4 Frisch, M. J., Trucks, G. W., Schlegel, H. B., Scuseria, G. E., Robb, M. A., Cheeseman, J. R., Scalmani, G., Barone, V., Mennucci, B., Petersson, G. A., Nakatsuji, H., Caricato, M., Li, X., Hratchian, H. P., Izmaylov, A. F., Bloino, J., Zheng, G., Sonnenberg, J. L., Hada, M., Ehara, M., Toyota, K., Fukuda, R., Hasegawa, J., Ishida, M., Nakajima, T., Honda, Y., Kitao, O., Nakai, H., Vreven, T., Montgomery, J. A., Peralta, J. E., Ogliaro, F., Bearpark, M., Heyd, J. J., Brothers, E., Kudin, K. N., Staroverov, V. N., Kobayashi, R., Normand, J., Raghavachari, K., Rendell, A., Burant, J. C., Iyengar, S. S., Tomasi, J., Cossi, M.,

Rega, N., Millam, J. M., Klene, M., Knox, J. E., Cross, J. B., Bakken, V., Adamo, C., Jaramillo, J., Gomperts, R., Stratmann, R. E., Yazyev, O., Austin, A. J., Cammi, R., Pomelli, C., Ochterski, J. W., Martin, R. L., Morokuma, K., Zakrzewski, V. G., Voth, G. A., Salvador, P., Dannenberg, J. J., Dapprich, S., Daniels, A. D., Farkas, Ö., Foresman, J. B., Ortiz, J. V., Cioslowski, J. & Fox, D. J. Gaussian 09, Revision B.01; Gaussian, Inc.: Wallingford, CT, 2009.

S5 Grimme, S. Semiempirical GGA-type density functional constructed with a long-range dispersion correction. J*. Comput. Chem*. **27**, 1787– 1799 (2006).

S6 Grimme, S. Accurate description of van der Waals complexes by density functional theory including empirical corrections. *J. Comput. Chem*. **25**, 1463 – 1473 (2004).

S7 Dunning, T. H. Jr. Gaussian basis sets for use in correlated molecular calculations. I. The atoms boron through neon and hydrogen. *J. Chem. Phys*. **90**, 1007-1023 (1989)

S8 Barone, V. & Cossi, M. Quantum calculation of molecular energies and energy gradients in solution by a conductor solvent model. *J. Phys.Chem. A.* **102**, 1995-2001 (1998).

S9 Improta, R., Barone, V., Scalmani, G. & Frisch, M. A state-specific polarizable continuum model time dependent density functional theory method for excited-state calculations in solution. *J. Chem. Phys.* **125**, 054103-9 (2006).
